# An Angiopep2-PAPTP Construct Overcomes the Blood-Brain Barrier. New Perspectives against Brain Tumors

**DOI:** 10.3390/ph14020129

**Published:** 2021-02-06

**Authors:** Sofia Parrasia, Andrea Rossa, Tatiana Varanita, Vanessa Checchetto, Riccardo De Lorenzi, Mario Zoratti, Cristina Paradisi, Paolo Ruzza, Andrea Mattarei, Ildikò Szabò, Lucia Biasutto

**Affiliations:** 1 Department of Biomedical Sciences, University of Padova, Viale G. Colombo 3, 35131 Padova, Italy; sofia.parrasia@phd.unipd.it (S.P.); zoratti@mail.bio.unipd.it (M.Z.); 2 Department of Chemical Sciences, University of Padova, Via F. Marzolo 1, 35131 Padova, Italy; andrea1rossa@gmail.com (A.R.); delo9793@gmail.com (R.D.L.); cristina.paradisi@unipd.it (C.P.); paolo.ruzza@unipd.it (P.R.); 3 Department of Biology, University of Padova, Viale G. Colombo 3, 35131 Padova, Italy; tatiana.varanita@unipd.it (T.V.); vanessa.checchetto@unipd.it (V.C.); ildi@mail.bio.unipd.it (I.S.); 4 CNR Institute of Biomolecular Chemistry, Via F. Marzolo 1, 35131 Padova, Italy; 5 CNR Neuroscience Institute, Viale G. Colombo 3, 35131 Padova, Italy; 6 Department of Pharmaceutical and Pharmacological Sciences, University of Padova, Via F. Marzolo 5, 35131 Padova, Italy; andrea.mattarei@unipd.it

**Keywords:** PAPTP, Angiopep-2, blood-brain barrier, brain delivery, glioma

## Abstract

A developing family of chemotherapeutics—derived from 5-(4-phenoxybutoxy)psoralen (PAP-1)—target mitochondrial potassium channel mtKv1.3 to selectively induce oxidative stress and death of diseased cells. The key to their effectiveness is the presence of a positively charged triphenylphosphonium group which drives their accumulation in the organelles. These compounds have proven their preclinical worth in murine models of cancers such as melanoma and pancreatic adenocarcinoma. In in vitro experiments they also efficiently killed glioblastoma cells, but in vivo they were powerless against orthotopic glioma because they were completely unable to overcome the blood-brain barrier. In an effort to improve brain delivery we have now coupled one of these promising compounds, PAPTP, to well-known cell-penetrating and brain-targeting peptides TAT_48–61_ and Angiopep-2. Coupling has been obtained by linking one of the phenyl groups of the triphenylphosphonium to the first amino acid of the peptide via a reversible carbamate ester bond. Both TAT_48–61_ and Angiopep-2 allowed the delivery of 0.3–0.4 nmoles of construct per gram of brain tissue upon intravenous (i.v.) injection of 5 µmoles/kg bw to mice. This is the first evidence of PAPTP delivery to the brain; the chemical strategy described here opens the possibility to conjugate PAPTP to small peptides in order to fine-tune tissue distribution of this interesting compound.

## 1. Introduction

**Figure 1:**
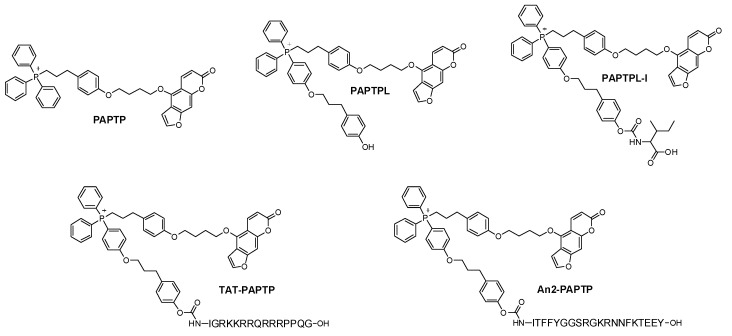
Structure of the major compounds synthesized.

Mitochondria are emerging as a prime target in oncology [1,2,3,4,5]. A contribution to progress in this direction has been the development of mitochondriotropic variants [6] of the psoralenic compound 5-(4-phenoxybutoxy)psoralen (PAP-1) [7,8]. These compounds, (3-(4-(4-((7-oxo-7*H*-furo[3,2-g]benzopyran-4-yl)oxy)butoxy)phenyl)propyl) triphenyl phosphonium iodide (PAPTP) and (3-(((4-(4-((7-oxo-7*H*-furo[3,2-g]chromen-4-yl)oxy) butoxy)phenoxy)carbonyl) amino)propyl)triphenylphosphonium iodide (PCARBTP) (Figure 1 and Figure S1), concentrate in the organelles, bind to and inhibit the K^+^ channel Kv1.3 present in the inner mitochondrial membrane (IMM) [9], interact with Complex I of the respiratory chain [10], and thereby cause oxidative stress which selectively kills cancer cells sparing normal ones [6]. Accumulation in mitochondria is determined by the presence in the molecule of a lipophilic permanent cation (in this case, as most often, triphenylphosphonium (TPP) [11,12]), and is essential. The parent compounds not containing this mitochondrial “address” are vastly less powerful, since inhibition of only the Kv1.3 population residing in the plasma membrane inhibits proliferation [13,14] but does not result in cell death. These novel “mitocans” are effective in vitro against a variety of cancer cells expressing the channel. *In vivo* they are currently being studied, in particular, in murine models of melanoma, pancreatic ductal adenocarcinoma (PDAC) and chronic lymphocytic leukemia (CLL) [6,15].

PAPTP and PCARBTP efficiently killed cultured glioma and glioblastoma cell lines but did not affect tumor growth in an orthotopic murine model [16]. The failure can be attributed to the inability to permeate the blood-brain barrier (BBB) since the drugs have been shown to reach various organs, but not the brain [6]. In turn, this difficulty may be due at least in part precisely to the TPP group, since the parent compound, PAP-1, is effective against various Central Nervous System (CNS) disorders [17,18,19,20]. This difficulty is not surprising if one compares the characteristics of these molecules to the parameters found to favour/hinder BBB permeation [21,22,23,24].

One possibility to extend the range of possible applications for these compounds is to facilitate access to the brain by reversibly conjugating PAPTP to a molecular “ferry”, i.e., a molecule capable to cross the BBB, hopefully taking the active specie along. The goal is to achieve delivery of these compounds to intracerebral cancers.

Some cell-penetrating peptides are reported to function as trans-BBB “ferries” of this type [25,26,27,28,29,30]. The first, possibly best-known and one of the most efficient [31,32] of these is TAT, a segment of the transactivator of transcription of human immunodeficiency virus (HIV) [33,34]. TAT has been shown to penetrate the BBB in a non-facilitated process [31,34]. Angiopep-2 (An2), derived from aprotinin, has emerged more recently as a valid alternative [35,36,37,38]. Importantly, this peptide exploits the low density lipoprotein receptor related protein-1 (LRP-1)—expressed both by brain microcapillary endothelial cells and by glioma—to gain access to the brain parenchyma via receptor-mediated transcytosis [39].

Both peptides have already been extensively used in efforts to deliver drugs to gliomas [25,27]. ANG1005, a drug composed of paclitaxel covalently linked to An2, has been tested against brain metastases of breast cancer [40,41] and is undergoing other clinical trials.

As reported here, these peptides have, therefore, been used to build reversible conjugates comprising PAPTP, to verify whether these constructs would be targeted to the brain in the mouse. A previous recent study [10] has identified the contributions of the component sections of PAP-1 to its pharmacological activity. The compound binds to mtKv1.3—which is physically associated with complex I of the respiratory chain—using the alkoxyphenoxy “arm”. This binding positions the coumarinic (psoralenic) ring system so that it can accept electrons from the respiratory complex, diverting them to molecular oxygen to generate reactive oxygen species (ROS), which are the instrument of cell death. Therefore, the peptide chain could not be attached to any other position than one of the phenyl rings of the triphenylphosphonium group.

## 2. Results

### 2.1. Preliminary Studies

PCARBTP itself contains an hydrolyzable carbamate function, whose presence may complicate analysis and pharmacokinetics. More stable PAPTP was thus chosen as the active principle to be delivered to the brain in this proof-of-principle study. In order to interfere as little as possible with binding to and inhibition of Kv1.3, the peptide component of the construct (the *BBB-ferry*) was linked to one of the phenyl rings of the triphenylphosphonium group, which was functionalized for the purpose with an OH group in position 4 (structure PAPTPOH in Figure S1). The original idea was to conjugate the selected *BBB-ferry* directly to this hydroxyl via a carbamate ester group, a linkage which often shows the proper reactivity and releases the active principle within a useful time window in physiological environments [42]. The reactivity of a phenolic carbamate ester bearing a para phosphonium substituent—and thus activated for hydrolysis—was preliminarily tested using a simpler model which was synthesized for the purpose (Figure S1, Model S1). Experiments with this model proved that at physiological pH values such carbamate esters undergo hydrolysis too rapidly to be useful. Thus, it was necessary to insert a distancing short linker terminating with a phenolic hydroxyl to be used to build more stable carbamate derivatives (Figure S1, Model S2). This approach produced the desired effect, and was thus applied to PAPTPOH (structure PAPTPL in Figure 1). Furthermore, an amino acid (isoleucine) was added as part of the synthetic strategy (PAPTPL-I in Figure 1). Its presence allowed the direct coupling of the construct to the peptide still attached to the solid-phase-synthesis (Wang) resin via a peptide bond. Thus, the molecule to be linked to *BBB-ferry* peptides took the form of PAPTPL-I and was used to develop the two constructs used in this study, TAT-PAPTP and An2-PAPTP (Figure 1).

### 2.2. Synthesis of PAPTPL-I, TAT-PAPTP, and An2-PAPTP

#### 2.2.1. Synthesis of PAPTPL-I

**Scheme 1:**
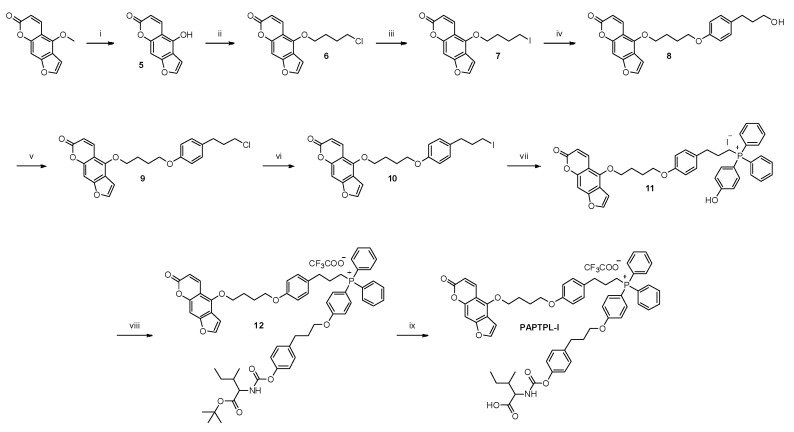
Synthesis of PAPTPL-I. Reagents and conditions: (**i**) BBr_3_, DCM, r.t. for 100 min, 98% yield; (**ii**) 1-bromo-4-chlorobutane, Cs_2_CO_3_, N,N-dimethylformamide (DMF), 50 °C overnight, 87% yield; (**iii**) NaI, acetone, 70 °C overnight, 87% yield; (**iv**) 3-(4-hydroxyphenyl)-1-propanol, Cs_2_CO_3_, DMF, 50 °C overnight, 83% yield; (**v**) SOCl_2_, pyridine, dichloromethane (DCM), 50 °C for 5.5 h, 98% yield; (**vi**) NaI, acetone, 70 °C overnight, 99% yield; (**vii**) (4-hydroxyphenyl)diphenylphosphine, toluene, 110 °C for 4 days, 69% yield; (**viii**) **4**, Et_3_N, DMF, 80 °C for 5 h, 23% yield; (**ix**) DCM/trifluoroacetic acid (TFA) 1:1, r.t. for 2 h, 91% yield.

**Scheme 2:**
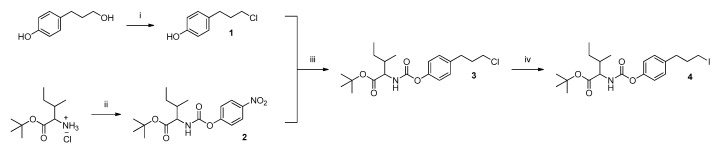
Synthesis of intermediate **4**. Reagents and conditions: (**i**) HCl 37%, 100 °C overnight, 99% yield; (**ii**) bis(4-nitrophenyl) carbonate, 4-dimethylaminopyridine (DMAP), DCM, 50 °C overnight, 76% yield; (**iii**) DMAP, DMF, 50 °C overnight, 61% yield; (**iv**) NaI, acetone, 60 °C overnight, 79% yield.

The synthesis of PAPTPL-I is illustrated in Scheme 1. It is a nine-step procedure starting from the naturally-occurring compound 5-methoxypsoralen. Four other reaction steps (Scheme 2) are required to produce intermediate **4**, used in step **viii**. The reaction conditions of the first three steps were as described in [6] and the yields were similar. Step **iv** is the nucleophilic substitution of iodine in intermediate **7** by 3-(4-hydroxyphenyl)-1-propanol. The hydroxyl group in intermediate **8** is replaced by chlorine in step **v** by reaction with thionyl chloride. Intermediate **9** is then converted to **10** by the Finkelstein reaction. The next step is the addition to the molecular structure of the diphenyl(p-OH-phenyl)phosphonium moiety. The hydroxyl is used to attach the linker to intermediate **11** in step **viii**, which consists in the reaction of **11** with **4** in the presence of triethylamine (TEA). This step is the most problematic one within the synthesis because, simultaneously with the formation of **12**, hydrolysis of the carbamate ester also occurs. This side reaction lowers the yield of **12**, but forms PAPTPL which was isolated and used for cytotoxicity assays and for the determination of recovery yields from tissues. Finally, in step **ix**, the *tert*-butyl ester in **12** is hydrolyzed in trifluoroacetic acid/dichloromethane (TFA/DCM) 1:1 to afford PAPTPL-I. The carboxyl acid deprotected in this step will be the junction point with the peptides.

#### 2.2.2. Synthesis of TAT-PAPTPL

TAT_48–61_ (GRKKRRQRRRPPQG) was initially chosen as the *BBB-ferry*. The peptide was synthesized by standard solid-phase synthetic procedures and coupled to PAPTPL-I while still attached to the Wang resin. After detachment, the construct was purified by preparative high-performance liquid chromatography (HPLC) (see Section 4.1.2 and Section 4.2.4). Its characterization by circular dichroism (CD) spectroscopy is presented in Supplementary Figures S2 and S3.

#### 2.2.3. Synthesis of An2-PAPTPL

This construct was synthesized following the same procedure used for TAT-PAPTP, by coupling PAPTPL-I with the An2 peptide (TFFYGGSRGKRNNFKTEEY) “grown” in Wang resin (not shown). Its characterization by CD spectroscopy is presented in Supplementary Figures S4 and S5.

### 2.3. Cytotoxicity of PAPTP-Linker (PAPTPL)

**Figure 2:**
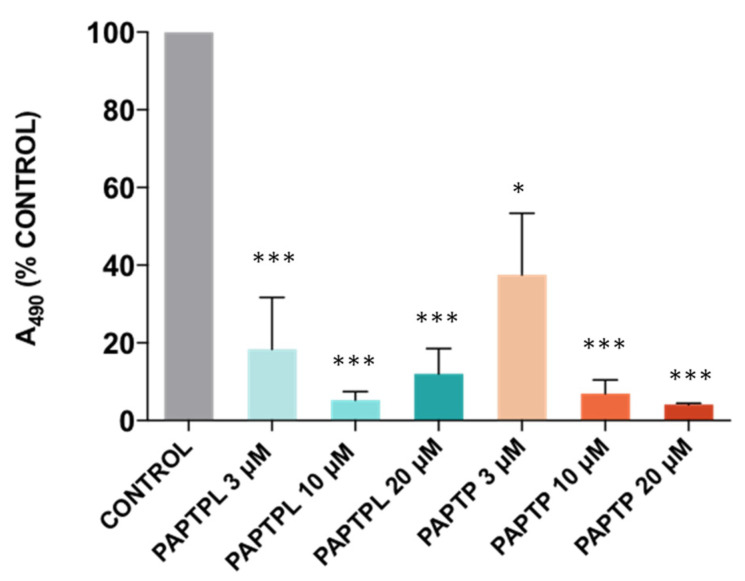
Cell viability of SHSY-5Y cells following treatment for 24 h with the indicated compounds (MTS assay, 490 nm). Values are reported as mean percentage ± standard error of the mean (SEM) of viable cells normalized with respect to untreated cells (*n* = 3); each condition was significantly different from control: * *p* < 0.05, *** *p* < 0.001, One-way analysis of variance (ANOVA).

Hydrolysis of the carbamate group in the peptidic construct will yield PAPTPL, which is expected to act as the active, cytotoxic, principle. Since the presence of the linker portion might alter the pharmacology of the molecule, the cytotoxicity of PAPTPL was compared with that of PAPTP using the neuroblastoma cell line SHSY-5Y, which expresses the mitochondrial channel Kv1.3. The results of the (3-(4,5-dimethylthiazol-2-yl)-5-(3-carboxymethoxyphenyl)-2-(4-sulfophenyl)-2*H*-tetrazolium) (MTS) reduction assay revealed that there is no statistically significant difference in the cytotoxic effects of PAPTP and PAPTPL (Figure 2). As expected on the basis of previous analogous tests on cancer cell lines, both compounds exhibited a marked cytotoxicity.

### 2.4. Aqueous Solubility

Figure 2 shows that there was little dose-dependence of the cytotoxic effects of PAPTP and PAPTPL. This unexpected observation may be attributed to the low solubility in aqueous media of these compounds, an important shortcoming of the family of psoralenic derivatives, including PAP-1 [10]. We thus determined the solubility of the major molecules of interest.

The solubility of PAPTP in 0.9% NaCl saline was 5.1 ± 0.8 μM, while that of PAPTPL was below the detection limit (0.1 µM). The solubility of TAT-PAPTP was >100 μM in water as well as in a saline solution.

An2-PAPTP precipitated in saline as well as in 10 mM phosphate buffer, and it could not be detected by HPLC analysis in the supernatants of the centrifugation protocol (see Section 4.4). Strikingly, its solubility in water turned out instead to be above 100 μM.

### 2.5. Stability in Blood

**Figure 3:**
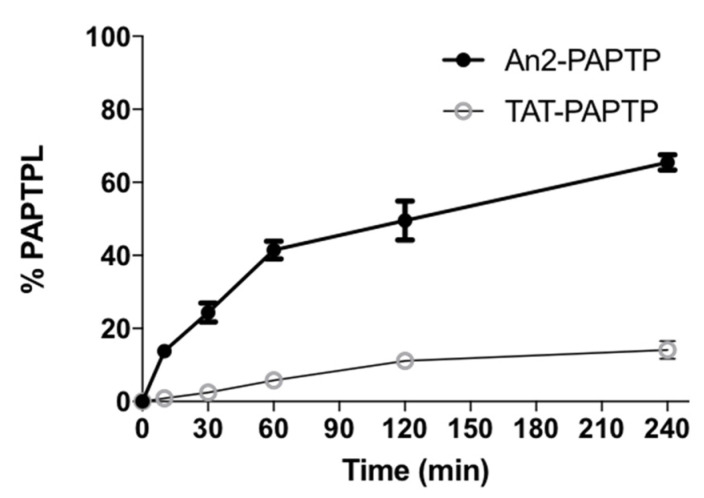
Kinetics of An2-PAPTP and TAT-PAPTP hydrolysis in mouse blood. Data refer to the percentage of PAPTPL released, and are expressed as mean ± SEM. *n* = 5 and 4, respectively.

An appropriate stability in the bloodstream is an important characteristic for any prodrug. TAT-PAPTP and An2-PAPTP ought to be stable enough to allow the penetration of the BBB before the release of the active PAPTPL. Upon incubation in fresh mouse blood at 37 °C, about 60% of An2-PAPTP was hydrolyzed over 4 h (Figure 3). TAT-PAPTP proved more stable (Figure 3).

Both conjugates could, therefore, be tested in vivo.

### 2.6. Brain Delivery of TAT-PAPTP

In order to choose the same administration vehicle for all the compounds to be tested and to avoid intravenous injection of precipitates (An2-PAPTP is insoluble in saline; see above, Section 2.4), the vehicle used for administrations was mouse plasma. We are not aware of other pharmacological studies utilizing plasma as a vehicle, but a precedent of sorts is provided by some rejuvenation or induced aging studies that examined the systemic effects of heterologous plasma administration [43,44]. TAT-PAPTP, An2-PAPTP and PAPTP did not form visible precipitates in plasma, presumably because they bind immediately to macromolecular components of the fluid.

TAT-PAPTP (5 µmoles/kg bw) was administered by tail vein injection to 2 C57BL mice. Unfortunately, the mice soon began to show evident signs of illness (respiratory difficulties, trailing limbs, swelling of the paws) and were sacrificed within 15 min as a humane experimental endpoint. The observed toxicity was not due to the use of plasma, since mice were not made ill by the administration of PAPTP or An2-PAPTP in the same vehicle (see below, Section 2.7).

The brains of these mice were analyzed to evaluate the presence of the construct in the tissue (see Section 4.7). Each brain was homogenized and incubated with an equal volume of phosphate-buffered saline (PBS, pH 12.5) for 24 h; 10 volumes of ethyl acetate were then added. After vigorous mixing, the organic phase was collected, concentrated and analyzed via HPLC. This procedure allows hydrolysis of the carbamate bond, leading to the extraction of PAPTPL, which is considered proportional to the amount of peptide-PAPTP conjugate (or its fragments) present in the brain. The results revealed that the construct was present at the level of 0.2–0.3 nmoles/g fresh tissue.

However, due to the toxic effects, experiments with TAT-PAPTP were discontinued in favor of tests with An2-PAPTP.

### 2.7. Tissue Distribution of An2-PAPTP

An2-PAPTP proved to be considerably less toxic than TAT-PAPTP, allowing the study of its distribution and of that of PAPTPL in organs as a function of time. In preliminary tests An2-PAPTP was administered at 5 µmol/kg bw by the intraperitoneal (*i.p.*) route, using dimethylsulfoxide (DMSO) as the vehicle. After sacrifice at 20 min or 1, 2, and 4 h after injection, no An2-PAPTP or PAPTPL could be detected in the brain of any of the four mice. Analysis of blood and liver (data not shown) suggested that only a small fraction of the drug had reached the circulation. Many brain-penetrating peptides bind to biomolecules such as proteins and cell structures such as membranes. Furthermore, the absence of a flowing bloodstream at the injection site may cause a poorly soluble molecule to remain confined in the vicinity of the point of delivery. Indeed, analysis of a sample collected from the peritoneum revealed that a large amount of An2-PAPTP had remained associated with that compartment (data not shown). Collectively, these preliminary results led to the adoption of the intravenous route. An2-PAPTP (5 µmoles/kg bw, in plasma) was thus administered by tail vein injection to mice.

#### 2.7.1. Brain Delivery of An2-PAPTP

An2-PAPTP was present in the brain at 15 and 30 min post-injection (see Table 1 for quantifications), while only a trace could be detected after one hour. Thus, An2-PAPTP could reach the brain and did not show toxicity, in contrast to TAT-PAPTP. 

PAPTP itself (5 µmoles/kg bw) was also administered to mice using the same procedure as for TAT-PAPTP and An2-PAPTP, but did not reach detectable levels in the brain of mice. This observation is in agreement with the results obtained by *i.p.* injection [6], and excludes that brain delivery of An2-PAPTP may be due to an increase in BBB permeability caused by administration of the vehicle (i.e., heterologous plasma) itself. No sign of toxicity was observed in these latter experiments, in agreement with previous results [6].

#### 2.7.2. Liver Metabolism of An2-PAPTP

**Figure 4:**
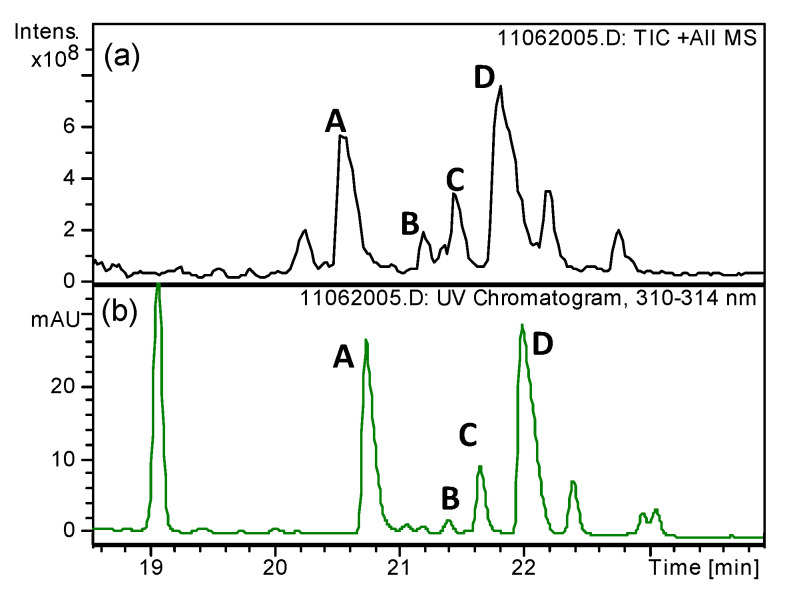
Representative high-performance liquid chromatography/mass spectrometry (HPLC/MS) analysis of a liver extract 1 h after administration of An2-PAPTP. (**a**) Total ion current chromatogram; (**b**) UV chromatogram, 310–314 nm. Identification of the main HPLC peaks: A = PAPTPL-IT (*m*/*z* = 1061.6), B = PAPTPL (*m*/*z* = 803.4), C = PAPTPL-I (*m*/*z* = 960.5), D = PAPTPL-ITF (*m*/*z* = 1208.7).

**Figure 5:**
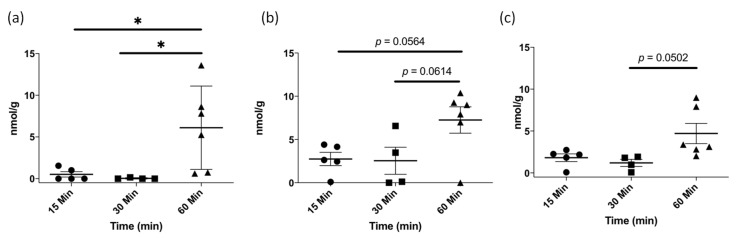
Concentrations of An2-PAPTP-derived metabolites in liver: (**a**) PAPTPL-I; (**b**) PAPTP-IT; (**c**) PAPTP-ITF. Data are expressed as mean ± SEM (*n* = 5, 4, and 6 for 15, 30 and 60 min, respectively). *: *p* < 0.05, One-way ANOVA.

Since the liver is involved in detoxification, generally accumulating major fractions of drugs administered via the bloodstream, liver was analyzed as well. As expected, the analyses showed that An2-PAPTP is mainly metabolized through the degradation of the peptide chain. In particular, PAPTPL-isoleucine (PAPTPL-I), PAPTPL-isoleucine- threonine (PAPTPL-IT), and PAPTPL-isoleucine-threonine-phenylalanine (PAPTPL-ITF) were identified by HPLC/MS (mass spectrometry) analysis (Figure 4). The levels of PAPTL-I increased significantly at one hour from the injection, in comparison to the levels measured at both 15 and 30 min. An analogous time-course was observed for PAPTPL-IT and PAPTPL-ITF, although the *p* values of the comparisons turned out to remain slightly above 0.05 (Table 2 and Figure 5). The amount of PAPTPL did not change significantly over time, ranging between 1.5 ± 0.6 nmol/g and 0.4 ± 0.2 nmol/g (Figure 5). The data suggest that liver peptidases degraded the peptide chain nearly completely in little more than one hour.

The total amount of An2-PAPTP and its metabolites, summarized in Table 3, is comprehensive of all the species having the characteristic psoralenic UV spectrum obtained by the overall extraction procedure (i.e., from organic solvent extraction and basic hydrolysis of the remaining pellet), and represents and underestimation of the real concentration in the tissue (see Section 4.7.3). This is because no recovery yield correction was applied, since it was impossible to identify most of the species extracted, and because PAPTPL extracted after high-pH hydrolysis of the pellet could come either from An2-PAPTP or from metabolites. For quantification in HPLC analysis, all the psoralenic species were assumed to have the same molar extinction coefficient as PAPTPL, an assumption justified by the near-identity observed for all the available psoralen-containing molecules.

#### 2.7.3. Levels of An2-PAPTP and Metabolites in Other Organs

To better characterize the tissue distribution profile of An2-PAPTP we analyzed also spleen, kidneys and heart (Table 4 and Supplementary Tables S1–S3). The same metabolites detected in the liver (i.e., PAPTPL, PAPTPL-I, PAPTPL-IT, PAPTPL-ITF) were also identified in the spleen. In all the analyzed organs the amount of the psoralenic species corresponded at most to 1.2% of the administered dose, with the highest concentrations found in the spleen. Only minor amounts of the detected analytes were found in the heart.

### 2.8. Angiopep2-PAPTP Does Not Disrupt Membranes

**Figure 6:**
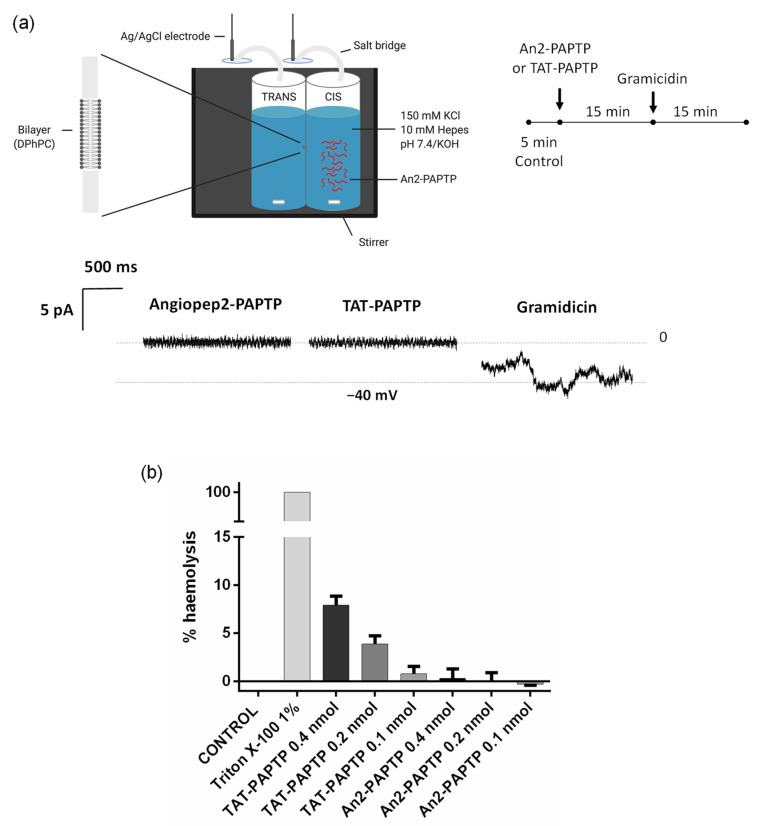
Effects of TAT-PAPTP and An2-PAPTP on membranes. (**a**) Electrophysiological analyses in planar lipid bilayers. Upper: schematic representations of the bilayer setup and of the experimental protocol used. Lower: exemplary current traces recorded after the addition of 0.5 µM An2-PAPTP (*n* = 9), 0.5 µM TAT-PAPTP (*n* = 2) and 2 µM gramicidin (as control, *n* = 3). All traces were recorded at −40 mV. (**b**) Haemolysis assay. Amounts of TAT-PATP and An2-PAPTP are expressed as nmoles/10^7^ erythrocytes. Mean values + SEM; *n* = 3.

As mentioned in the Introduction, Angiopep2 interacts with LRP-1 and mediates brain entry by transcytosis. There is no evidence in the literature of a membrane-permeabilizing effect similar to that of membrane-active peptides—such as, e.g., melittin [45,46]—which might lead to BBB disruption and leakiness. Since however each construct has its own, hard to predict, properties, An2-PAPTP and TAT-PAPTP were tested in electrophysiological planar bilayer experiments to verify whether they would cause the appearance of ion currents, i.e., membrane permeabilization. As shown in Figure 6, this was not the case for either construct. The planar membranes remained without exception “silent” after the addition of 0.5 µM compound, a concentration chosen to approximate the about 0.4 nmoles/gr of (mostly bound) psoralenic species found in the brain (see Section 4.10 for experimental details). Subsequent or parallel addition of a peptide known to form short-lived pores spanning phospholipid bilayers, gramicidin, led instead to the expected development of “fast” ion currents.

To further investigate the impact of these constructs on biomembranes we conducted haemolysis assays. Again, under the experimental conditions applied, An2-PAPTP did not induce a measurable release of haemoglobin from mouse Red Blood Cells (Figure 6). On the other hand TAT-PAPTP did, suggesting that haemolysis may contribute to its toxicity.

## 3. Discussion

The results reported above confirm that conjugation to either TAT or An2 can be an effective strategy to ferry reluctant drugs—even a relatively complex and large molecule like PAPTPL—across the BBB. To the best of our knowledge, this is the first application of this pro-drug strategy to a mitochondriotropic, triphenylphosphonium-containing drug. TPP-driven mitochondriotropic compounds are not necessarily excluded from the brain [47,48,49,50]. The literature contains several reports of the use of mitochondria-targeted probes to report on cerebral processes [51] or of mitochondria-targeted antioxidants to ameliorate neurodegeneration [52,53] or the consequences of brain trauma [54,55,56] or stroke [57,58]. In most cases, however, what was studied were the biochemical/patophysiological/behavioural consequences of drug administration, while the presence of the compound in the brain was essentially taken for granted. Among the exceptions to this statement are studies by the group of Murphy and Smith, using radioactively-labeled compounds [50,59]. Intravenous (i.v.) injection of 100 nmol ^3^H-triphenylmethylphosphonium (TPMP) in mice resulted in an initial peak concentration of about 0.3–2 nmol/gr brain tissue, as determined by scintillation counting. In the case of ^3^H-MitoQ, the value in wild-type (WT) mice was about 0.05 nmol/gr (Area Under Curve: 0.6 nmol·h/g wet weight) [59]. 10-day administration of 500 µM ^3^H-TPMP, ^3^H-MitoQ, or ^3^H-mitoVitE in drinking water produced steady-state brain radioactivity levels corresponding to about 9, 0.15, and 3.5 nmol/gr fresh tissue, respectively [50]. A conclusion reached was that uptake of MitoQ into the brain takes place to a lower extent than in other organs, due at least in part to the operation of multiple drug-resistance pumps at the BBB [59]. In a recent paper [60] MitoQ was quantified in the brain, liver and muscles of WT and R6/2 (a model of Huntington disease) mice which had drunk 500 µM MitoQ in water for 6 weeks. The study confirmed the amount in the brain to be much lower than in the other organs: about 1 pmol/g and 5 pmol/g for WT and R6/2 mice respectively. Thus, mitochondriotropic TPP-driven compounds, like most drugs, seem to penetrate the BBB with difficulty, whose degree varies from case to case and is evidently high in the case of PAPTP.

A second consideration may concern the efficacy of peptide-facilitated brain delivery. As mentioned in the introduction, there is ample evidence that conjugation to certain peptides, such as TAT and An2, may increase the amount/concentration of “cargo” molecules in the brain in comparison to administration of the “cargo” as such. Some papers also report information on the levels of active principle that can be achieved, and allow an estimate of the efficiency of delivery to the brain. In a relevant example, Thomas and colleagues injected ANG1005—the An2:paclitaxel 1:3 conjugate—i.v. into 20-g mice (14 nmol/g bw of radiolabeled ANG1005, equivalent to 42 nmol/g bw of paclitaxel (PTX)) and after 30 min found 0.62 nmol/g of ANG1005—calculated from radioactivity counts—in the parenchyma [61]. This in principle corresponds to 1.9 nmol/g of PTX, with a delivery to the brain of approximately 0.11% of the injected dose (assuming a 0.5-g brain). This still represented a 54-fold higher delivery of ANG1005 compared to an equimolar dosage of paclitaxel (and a 161-fold higher delivery of PTX when considering that each ANG1005 carries 3 molecules of paclitaxel). Analogous results were obtained using similar radioactively labelled conjugates carrying doxorubicin (ANG1007) or etoposide (ANG1009) instead of paclitaxel [62]. With these two constructs, assuming a 0.5-g brain, approximately 0.08% (ANG1007, doxorubicin) and 0.17% (ANG1009, etoposide) of the injected dose had found its way to the brain 30 min. after i.v. injection, again representing major increases when compared to administration of the active principles as such. Furthermore, the drug levels increased substantially in orthotopically implanted U-87 glioma masses.

These peptides have also been used to facilitate brain uptake of nanovehicles. Kadari and colleagues grafted An2 onto the surface of solid lipid docetaxel-loaded nanoparticles to deliver docetaxel (DTX) to the brain [63]. This doubled (vs. undecorated nanoparticles) the peak concentration of DTX found in brain, which reached 4.13 µg/g, corresponding to approximately 0.9% of the administered dose (10 µg/g bw; i.v.). Qin et al. (2011) covalently linked TAT to cholesterol, and used this construct to dope doxorubicin-loaded liposomes which were injected i.v. into mice at a total doxorubicin dose of 2.5 µg/g bw [64]. In this case the peak doxorubicin concentration in brain was about 0.45 µg/g, corresponding to a brain delivery of approximately 0.4% of the injected dose.

Injecting 5 nmol/g bw of An2-PAPTP (and TAT-PAPTP) the psoralenic species measured in the brain in the present study amounted to 0.1–0.2% of the injected dose, well in line with the performance of ANG1005/7/9.

This performance was achieved despite some difficulties due to the intrinsic chemico-physical characteristics of the compounds. One is the insolubility of An2-PAPTP in aqueous saline media. Although solubility in water is at least three orders of magnitude higher (>100 µM), it was still inadequate for the administration of the desired dose as aqueous solution. Furthermore, it is obviously not recommendable to inject water or organic solvents intravenously. A viable alternative was provided by mouse plasma. In this vehicle An2-PAPTP is expected to bind to soluble proteins, including albumin, thus avoiding precipitation and insuring transport along the bloodstream from the injection point. Evidence for such an association has been provided by Thomas and colleagues concerning ANG1005 [61]. The “sticky” character of these molecules, especially TAT-PAPTP, is proven by the difficulties we had to overcome to analyze tissues (see Section 4.7.2 and Section 4.7.3).

While in the name of “reduction” a proper toxicological study was not carried out, the toxicity of TAT-PAPTP was evident. Peptide toxicity issues are not often discussed in the literature, but peptides, in particular arginine-rich ones, can be toxic in vivo as well as for cultured cells [65]. The LD_50_ of TAT_47–57_ in mice has been reported as 17.5 µmol/kg bw [65]. Here the TAT-PAPTP conjugate was administered at 5 µmol/kg bw. However, conjugates can behave differently and have different toxicities depending on the attached “cargo” [54,65,66], as confirmed by the results of the present study, which show that TAT-PAPT is more toxic than its individual components (TAT and PAPTP) as well as than An2-PAPTP.

While the outcomes of this investigation are encouraging, it remains to be seen whether the achievable brain levels of PAPTP would be efficacious against, say, glioma. While therapy-oriented studies are worth considering, it may well be worthwhile to also explore other selective delivery approaches. One may be to test other peptide “ferries” [67]. An example may be L57 (TWPKHFDKHTFYSILKLGKH), which, like An2, binds LRP-1. The “brain input” for this ^125^I-labeled peptide one hour after i.v. administration was found to be approximately 0.04% of the injected dose [68], but advantages may derive from its low net positive charge and the absence of arginines, which should limit adhesion phenomena. Other possibilities are offered by the several nanovehicles that have been devised to target the brain and gliomas in particular. These include, e.g., the aforementioned one developed by Kadari et al. [63], or the nanomicelles functionalized with both Angiopep-2 and TAT described by Zhu et al. [69].

## 4. Materials and Methods

### 4.1. Chemicals and Instrumentation

HPLC-grade acetonitrile (ACN) was obtained from Carlo Erba (Milan, Italy) and used without further purification. Protected amino acids and pre-loaded Wang resins for solid state peptide synthesis, 2-(1*H*-benzotriazol-1-yl)-1,1,3,3-tetramethyluronium hexafluorophosphate (HBTU), 1-hydroxybenzotriazole (HOBt), 1-[Bis(dimethylamino) methylene]-1H-1,2,3-triazolo[4,5-b]pyridinium 3-oxide hexafluorophosphate (HATU) were purchased from Iris Biotech (Marktredwitz, Germany). 5-methoxypsoralen was purchased from Carbosynth. Other reagents and solvents were purchased from Sigma Aldrich (Milan, Italy), and were used as received. Thin-layer chromatography was performed on silica gel supported on plastic (Merck, Milan, Italy), and spots were visualized by UV illumination. Flash chromatography was performed on silica gel (Macherey-Nagel 60, 230–400 mesh granulometry (0.063–0.040 mm)) under air pressure.

#### 4.1.1. Nuclear Magnetic Resonance (NMR) Analysis

^1^H and ^13^C nuclear magnetic resonance (NMR) spectra were recorded with a Bruker 500 Avance III operating at 500 MHz (for ^1^H NMR) and 126 MHz (for ^13^C NMR) or with a Bruker 400 Avance III HD operating at 400/101 MHz. Chemical shifts (δ) are given in parts per million (ppm) relative to the signal of the solvent. The following abbreviations are used to indicate multiplicities: s, singlet; d, doublet; dd, doublet of doublets; t, triplet; m, multiplet; br, broad signal.

#### 4.1.2. Preparative High-Performance Liquid Chromatography (HPLC)

TAT-PAPTP and An2-PAPTP were purified using a Shimadzu SCL8A instrument, equipped with an LC8A double pump and an SPD-6A Prominence UV-Visible detector set at 216 nm, mounting a reversed-phase column (Vydac C18, 10 μm, 250 × 22 mm, 300 Å, Dionex, a division of ThermoFisher Scientific, Monza, Italy), at a flow rate of 12 mL/min.

Intermediate **12** (Scheme 1), PAPTPL, and PAPTPL-I were purified using a Shimadzu SCL8A instrument, equipped with an LC8A double pump and an SPD-20A Prominence UV-Visible detector set at 254 nm, mounting a reversed-phase column (Zorbax Eclipse XDB-C18 PrepHT, 5 μm, 150 × 21.2 mm, Agilent Technologies, Milan, Italy), at a flow rate of 17 mL/min.

Mobile phases and gradients used for the purification of these compounds are specified in the respective sections.

#### 4.1.3. HPLC-UV (Ultraviolet) Analysis

Quantitative analyses of solutions of the peptide-PAPTPs, PAPTPL, PAPTPL-I and of solutions resulting from the work-up of tissue samples were carried out by HPLC/UV (1290 Infinity LC System, Agilent Technologies, Milan, Italy using a reversed-phase column (Zorbax RRHT Extend C18, 1.8 µm, 50 × 3.0 mm, Agilent Technologies) and a UV diode array detector (190–500 nm), at a flow rate of 0.6 mL/min. Solvents A and B were water +0.1% TFA and ACN, respectively. The gradient for B was as follows: 10% for 1 min, then from 10 to 100% in 7.0 min, 100% for 0.5 min, then from 100% to 10% in 1.5 min. The injection volume was 5 µL. The eluate was preferentially monitored at 312 nm (corresponding to an absorbance maximum of psoralenic compounds). The temperature of the column was kept at 25 °C.

#### 4.1.4. HPLC-UV/Electrospray Ionization–Mass Spectrometry (ESI-MS) Analysis

Electrospray ionization-mass spectrometry (ESI-MS) analysis of the intermediates of PAPTPL-I synthesis was carried out with an 1100 Series Agilent Technologies system. The ESI source operated in full-scan positive ion mode, applying the following ESI parameters: nebulizer pressure 20 psi, dry gas flow 5 L/min, dry gas temperature 325 °C. The flow rate was 0.05 mL/min.

HPLC-UV/ESI-MS analysis of TAT-PAPTP, An2-PAPTP, and tissue extracts was carried out with the same instrument, using a reversed-phase column (Kinetex C18, 5 µm, 150 × 4.6 mm, 100 Å, Phenomenex) and a flow rate of 1.0 mL/min. Solvents A and B were water +0.1% formic acid and ACN +0.1% formic acid, respectively. The gradient for B was as follows: 3 min at 10%, from 10% to 100% in 27 min, then 100% for 2 min. The eluate was monitored at 216, 250, and 312 nm. MS analysis was performed with an ESI source operating in full-scan positive ion mode, applying the following ESI parameters: nebulizer pressure 50 psi, dry gas flow 8 L/min, dry gas temperature 350 °C.

#### 4.1.5. High-Resolution Mass Spectrometry (HRMS) Analysis

High-resolution mass spectrometry (HRMS) analysis was performed using a Waters Xevo G2-S QTof mass spectrometer. 10 µL of a 10 µM solution of the compound in ACN + 0.1% TFA were injected using the Fast Flow Injection modality. Mass spectra were recorded in the positive ion mode, using the following parameters: Capillary: 1.5 KV; Source temperature: 100 °C; Desolvation gas temperature: 350 °C; Cone gas: 10 L/h; Desolvation gas flow: 800 L/h.

### 4.2. Synthesis of TAT-PAPTP and An2-PAPTP

#### 4.2.1. Synthesis of Intermediate 4

##### Synthesis of 4-(3-chloropropyl)phenol (**1**)

3-(4-hydroxyphenyl)-1-propanol (2.07 g, 13.6 mmol) and HCl 37% (20 mL) were stirred overnight at 100 °C in a sealed vial. Dichloromethane (DCM) (50 mL) was then added to the reaction mixture, and the separated organic phase was washed with water (150 mL). The aqueous phase was extracted with DCM (3 × 50 mL), the combined organic layers were dried over Na_2_SO_4_ and the solvent was removed under reduced pressure. The residue was purified by flash chromatography (DCM as eluent) to afford **1** (2.28 g, 13.4 mmol, 99% yield) as a transparent oil. ^1^H NMR (400 MHz, CDCl_3_) δ 7.11–7.04 (m, 2H), 6.82–6.75 (m, 2H), 5.12 (s, 1H), 3.52 (t, *J* = 6.5 Hz, 2H), 2.72 (t, *J* = 7.4 Hz, 2H), 2.10–1.99 (m, 2H). ^13^C NMR (101 MHz, CDCl_3_) δ 153.8, 133.1, 129.8, 115.5, 44.4, 34.3, 31.9 (Figure S6).

##### Synthesis of tert-butyl 3-methyl-2-(((4-nitrophenoxy)carbonyl)amino)pentanoate (**2**)

L-isoleucine *tert*-butyl ester hydrochloride (1.52 g, 6.79 mmol, 1.0 eq), bis(4-nitrophenyl) carbonate (2.07 g, 6.80 mmol, 1.0 eq) and 4-DiMethylAminoPyridine (DMAP) (1.68 g, 13.8 mmol, 2.0 eq) were dissolved in DCM (20 mL). The reaction mixture was stirred overnight at 50 °C, then it was diluted with DCM (50 mL) and washed with HCl 0.5 M (100 mL). The aqueous layer was extracted with DCM (3 × 50 mL), the combined organic layers were dried over Na_2_SO_4_ and the solvent was removed under reduced pressure. The residue was purified by flash chromatography (DCM/petroleum ether 8:2 as eluent) to afford **2** (1.83 g, 5.19 mmol, 76% yield) as a transparent oil. ^1^H NMR (500 MHz, CDCl_3_) δ 8.20 (d, *J* = 9.1 Hz, 2H), 7.30 (d, *J* = 9.1 Hz, 2H), 5.85 (d, *J* = 8.6 Hz, 1H), 4.26 (dd, *J* = 8.7, 4.4 Hz, 1H), 1.98–1.87 (m, 1H), 1.48 (s, 9H), 1.53–1.42 (m, 1H), 1.29–1.18 (m, 1H), 0.99–0.91 (m, 6H). ^13^C NMR (126 MHz, CDCl_3_) δ 170.6, 156.0, 153.0, 144.8, 125.1, 122.0, 82.7, 58.9, 38.4, 28.1, 25.2, 15.5, 11.8 (Figure S7). ESI-MS (*m*/*z*): 375.2 [M + Na]^+^.

##### Synthesis of tert-butyl 2-(((4-(3-chloropropyl)phenoxy)carbonyl)amino)-3-methylpentanoate (**3**)

**1** (114 mg, 668 µmol, 1.5 eq), **2** (157 mg, 446 µmol, 1.0 eq) and DMAP (109 mg, 892 µmol, 2.0 eq) were dissolved in anhydrous N,N-DiMethylFormamide (DMF) (3.0 mL). The reaction mixture was stirred overnight at 50 °C, then it was diluted with ethyl acetate (EtOAc) (100 mL) and washed with HCl 0.5 M/brine 1:1 (4 × 50 mL). The organic layer was dried over Na_2_SO_4_ and the solvent was removed under reduced pressure. The residue was purified by flash chromatography (DCM as eluent) to afford **3** (105 mg, 274 µmol, 61% yield) as a transparent oil. ^1^H NMR (500 MHz, CDCl_3_) δ 7.17 (d, *J* = 8.4 Hz, 2H), 7.05 (d, *J* = 8.4 Hz, 2H), 5.61 (d, *J* = 8.6 Hz, 1H), 4.28 (dd, *J* = 8.7, 4.4 Hz, 1H), 3.51 (t, *J* = 6.4 Hz, 2H), 2.76 (t, *J* = 7.4 Hz, 2H), 2.10–2.01 (m, 2H), 1.97–1.88 (m, 1H), 1.49 (s, 9H), 1.53–1.43 (m, 1H), 1.29–1.18 (m, 1H), 1.00–0.91 (m, 6H). ^13^C NMR (126 MHz, CDCl_3_) δ 171.0, 154.6, 149.4, 137.8, 129.4, 121.7, 82.4, 58.8, 44.2, 38.5, 34.1, 32.2, 28.2, 25.3, 15.5, 11.9 (Figure S8). ESI-MS (*m*/*z*): 328.2 [M-tBu + 2H]^+^.

##### Synthesis of tert-butyl 2-(((4-(3-iodopropyl)phenoxy)carbonyl)amino)-3-methylpentanoate (**4**)

**3** (918 mg, 2.39 mmol) was dissolved in acetone (10 mL) and NaI was added until saturation. The reaction mixture was stirred overnight at 60 °C, then it was diluted with DCM (50 mL) and washed with water (200 mL). The aqueous layer was extracted with DCM (2 × 50 mL), the combined organic layers were dried over MgSO_4_ and the solvent was removed under reduced pressure. The residue was purified by flash chromatography (first column: petroleum ether/acetone 10:1 as eluent; second column: DCM/petroleum ether/acetone 50:47:3 as eluent) to afford **4** (893 mg, 1.88 mmol, 79% yield) as a transparent oil. ^1^H NMR (500 MHz, CDCl_3_) δ 7.17 (d, *J* = 8.4 Hz, 2H), 7.05 (d, *J* = 8.4 Hz, 2H), 5.60 (d, *J* = 8.6 Hz, 1H), 4.27 (dd, *J* = 8.7, 4.4 Hz, 1H), 3.16 (t, *J* = 6.8 Hz, 2H), 2.71 (t, *J* = 7.3 Hz, 2H), 2.14–2.06 (m, 2H), 1.97–1.87 (m, 1H), 1.49 (s, 9H), 1.54–1.42 (m, 1H), 1.31–1.17 (m, 1H), 1.00–0.91 (m, 6H). ^13^C NMR (126 MHz, CDCl_3_) δ 170.9, 154.6, 149.5, 137.5, 129.4, 121.7, 82.3, 58.8, 38.5, 35.6, 34.9, 28.2, 25.3, 15.5, 11.9, 6.4 (Figure S9). ESI-MS (*m*/*z*): 498.2 [M + Na]^+^, 514.1 [M + K]^+^.

#### 4.2.2. Synthesis of Intermediate PAPTPL-I

##### Synthesis of 4-hydroxy-7H-furo[3,2-g]chromen-7-one (**5**)

A 1.0 M BBr_3_ solution in DCM (69.4 mL, 69.4 mmol, 5.0 eq) was slowly added to a stirred 5-methoxypsoralen (3.00 g, 13.9 mmol, 1.0 eq) solution in DCM (140 mL) under nitrogen. The reaction mixture was stirred for 100 min at room temperature, then it was washed with saturated aqueous NaHCO_3_ (500 mL). The aqueous layer was extracted with DCM (300 mL), then with EtOAc (400 mL). The combined organic layers were dried over Na_2_SO_4_ and the solvent was removed under reduced pressure to afford **5** (2.75 g, 13.6 mmol, 98% yield) as an off-white solid [6]. ^1^H NMR (500 MHz, DMSO-*d*_6_) δ 8.23 (d, *J* = 9.6 Hz, 1H), 7.88 (d, *J* = 2.3 Hz, 1H), 7.18 (dd, *J* = 2.2, 0.9 Hz, 1H), 7.12 (s, 1H), 6.23 (d, *J* = 9.7 Hz, 1H). ^13^C NMR (126 MHz, DMSO) δ 160.5, 157.1, 152.7, 148.1, 145.0, 139.9, 112.6, 110.9, 104.8, 103.8, 91.0 (Figure S10). ESI-MS (*m*/*z*): 203 [M + H]^+^.

##### Synthesis of 4-(4-chlorobutoxy)-7H-furo[3,2-g]chromen-7-one (**6**)

We dissolved **5** (2.75 g, 13.6 mmol, 1.0 eq) and 1-bromo-4-chlorobutane (3.50 g, 20.4 mmol, 1.5 eq) in anhydrous DMF (100 mL). Cs_2_CO_3_ (6.65 g, 20.4 mmol, 1.5 eq) was added and the suspension was stirred overnight at 50 °C. Then the reaction mixture was diluted with EtOAc (600 mL) and washed first with 0.5 M HCl (400 mL), then with water (400 mL). The organic layer was dried over Na_2_SO_4_ and the solvent was removed under reduced pressure. The residue was purified by flash chromatography (DCM/EtOAc 98:2 as eluent) to afford **6** (3.46 g, 11.8 mmol, 87% yield) as a white solid [6]. ^1^H NMR (500 MHz, CDCl_3_) δ 8.11 (d, *J* = 9.7 Hz, 1H), 7.58 (d, *J* = 2.4 Hz, 1H), 7.11–7.10 (m, 1H), 6.93 (dd, *J* = 2.4, 0.9 Hz, 1H), 6.26 (d, *J* = 9.8 Hz, 1H), 4.49 (t, *J* = 5.8 Hz, 2H), 3.66 (t, *J* = 6.0 Hz, 2H), 2.10–2.00 (m, 4H). ^13^C NMR (126 MHz, CDCl_3_) δ 161.3, 158.4, 152.8, 148.8, 145.0, 139.3, 113.3, 112.8, 106.8, 105.2, 94.1, 72.1, 44.6, 29.3, 27.5 (Figure S11). ESI-MS (*m*/*z*): 293 [M + H]^+^.

##### Synthesis of 4-(4-iodobutoxy)-7H-furo[3,2-g]chromen-7-one (**7**)

We dissolved **6** (3.46 g, 11.8 mmol, 1.0 eq) in acetone (175 mL) and NaI (17.7 g, 118 mmol, 10 eq) was added. The reaction mixture was stirred overnight at 70 °C, then the solvent was removed under reduced pressure. The residue was taken up in EtOAc (350 mL) and washed with water (250 mL). The aqueous layer was extracted with EtOAc (200 mL), the combined organic layers were dried over Na_2_SO_4_ and the solvent was removed under reduced pressure. The residue was purified by flash chromatography (DCM/EtOAc 98:2 as eluent) to afford **7** (3.96 g, 10.3 mmol, 87% yield) as a white solid [6]. ^1^H NMR (500 MHz, CDCl_3_) δ 8.11 (d, *J* = 9.8 Hz, 1H), 7.58 (d, *J* = 2.4 Hz, 1H), 7.11–7.09 (m, 1H), 6.92 (dd, *J* = 2.4, 0.9 Hz, 1H), 6.26 (d, *J* = 9.8 Hz, 1H), 4.47 (t, *J* = 6.0 Hz, 2H), 3.28 (t, *J* = 6.6 Hz, 2H), 2.12–2.05 (m, 2H), 2.05–1.97 (m, 2H). ^13^C NMR (126 MHz, CDCl_3_) δ 161.3, 158.3, 152.8, 148.8, 145.0, 139.3, 113.3, 112.8, 106.7, 105.1, 94.1, 71.7, 31.0, 30.0, 6.0 (Figure S12). ESI-MS (*m*/*z*): 385 [M + H]^+^, 407 [M + Na]^+^.

##### Synthesis of 4-(4-(4-(3-hydroxypropyl)phenoxy)butoxy)-7H-furo[3,2-g]chromen-7-one (**8**)

We dissolved **7** (100 mg, 260 µmol, 1.0 eq) and 3-(4-hydroxyphenyl)-1-propanol (47.5 mg, 312 µmol, 1.2 eq) in anhydrous DMF (3.0 mL). Cs_2_CO_3_ (127 mg, 390 µmol, 1.5 eq) was added and the suspension was stirred overnight at 50 °C. Then the reaction mixture was diluted with EtOAc (100 mL) and washed with 0.5 M HCl (5 × 50 mL). The organic layer was dried over Na_2_SO_4_ and the solvent was removed under reduced pressure. The residue was purified by flash chromatography (DCM/acetone 9:1 as eluent) to afford **8** (88.0 mg, 215 µmol, 83% yield) as a white solid. ^1^H NMR (400 MHz, CDCl_3_) δ 8.12 (d, *J* = 9.8 Hz, 1H), 7.57 (d, *J* = 2.4 Hz, 1H), 7.14–7.07 (m, 3H), 6.95 (dd, *J* = 2.3, 0.8 Hz, 1H), 6.83–6.78 (m, 2H), 6.22 (d, *J* = 9.8 Hz, 1H), 4.54 (t, *J* = 6.0 Hz, 2H), 4.05 (t, *J* = 5.8 Hz, 2H), 3.70–3.62 (m, 2H), 2.68–2.61 (m, 2H), 2.13–1.98 (m, 4H), 1.91–1.81 (m, 2H), 1.48–1.39 (m, 1H). ^13^C NMR (101 MHz, CDCl_3_) δ 161.4, 158.4, 157.1, 152.8, 149.0, 144.9, 139.5, 134.2, 129.5, 114.5, 113.3, 112.6, 106.8, 105.3, 94.0, 72.7, 67.4, 62.4, 34.5, 31.3, 27.1, 26.1 (Figure S13). ESI-MS (*m*/*z*): 409.2 [M + H]^+^, 431.2 [M + Na]^+^, 447.1 [M + K]^+^.

##### Synthesis of 4-(4-(4-(3-chloropropyl)phenoxy)butoxy)-7H-furo[3,2-g]chromen-7-one (**9**)

We dissolved **8** (517 mg, 1.27 mmol, 1.0 eq) in anhydrous DCM (9.5 mL). Pyridine (103 µL, 1.27 mmol, 1.0 eq) and SOCl_2_ (184 µL, 2.54 mmol, 2.0 eq) were added and the solution was stirred for 5.5 h at 50 °C. Then the reaction mixture was diluted with DCM (100 mL) and washed with 0.5 M HCl (130 mL). The aqueous layer was extracted with DCM (2 × 60 mL), the combined organic layers were dried over Na_2_SO_4_ and the solvent was removed under reduced pressure. The residue was purified by flash chromatography (DCM/acetone 99:1 as eluent) to afford **9** (532 mg, 1.25 mmol, 98% yield) as a white solid. ^1^H NMR (500 MHz, CDCl_3_) δ 8.14 (d, *J* = 9.7 Hz, 1H), 7.58 (d, *J* = 2.4 Hz, 1H), 7.14–7.12 (m, 1H), 7.12–7.08 (m, 2H), 6.96 (dd, *J* = 2.4, 0.9 Hz, 1H), 6.84–6.80 (m, 2H), 6.24 (d, *J* = 9.8 Hz, 1H), 4.54 (t, *J* = 6.1 Hz, 2H), 4.05 (t, *J* = 5.8 Hz, 2H), 3.51 (t, *J* = 6.5 Hz, 2H), 2.72 (t, *J* = 7.4 Hz, 2H), 2.13–2.00 (m, 6H). ^13^C NMR (126 MHz, CDCl_3_) δ 161.4, 158.4, 157.3, 152.9, 149.0, 145.0, 139.4, 133.1, 129.7, 114.6, 113.3, 112.7, 106.8, 105.3, 94.0, 72.7, 67.4, 44.3, 34.3, 31.9, 27.1, 26.1 (Figure S14). ESI-MS (*m*/*z*): 427.2 [M + H]^+^, 449.2 [M + Na]^+^, 465.1 [M + K]^+^.

##### Synthesis of 4-(4-(4-(3-iodopropyl)phenoxy)butoxy)-7H-furo[3,2-g]chromen-7-one (**10**)

We dissolved **9** (622 mg, 1.46 mmol, 1.0 eq) in acetone (80 mL) and NaI (13.0 g, 86.7 mmol, 59 eq) was added. The reaction mixture was stirred overnight at 70 °C, then the solvent was removed under reduced pressure. The residue was taken up with DCM (100 mL) and washed with water (150 mL). The aqueous layer was extracted with DCM (2 × 70 mL), the combined organic layers were dried over Na_2_SO_4_ and the solvent was removed under reduced pressure. The residue was purified by flash chromatography (DCM/acetone 99:1 as eluent) to afford **10** (751 mg, 1.45 mmol, 99% yield) as a white solid [6]. ^1^H NMR (400 MHz, CDCl_3_) δ 8.14 (d, *J* = 9.8 Hz, 1H), 7.58 (d, *J* = 2.2 Hz, 1H), 7.19–7.05 (m, 3H), 6.99–6.93 (m, 1H), 6.82 (d, *J* = 8.4 Hz, 2H), 6.24 (d, *J* = 9.8 Hz, 1H), 4.54 (t, *J* = 5.9 Hz, 2H), 4.05 (t, *J* = 5.7 Hz, 2H), 3.15 (t, *J* = 6.8 Hz, 2H), 2.67 (t, *J* = 7.2 Hz, 2H), 2.16–1.97 (m, 6H). ^13^C NMR (101 MHz, CDCl_3_) δ 161.4, 158.4, 157.4, 152.8, 149.0, 144.9, 139.4, 132.8, 129.7, 114.6, 113.3, 112.7, 106.8, 105.3, 94.0, 72.7, 67.4, 35.4, 35.2, 27.1, 26.1, 6.5 (Figure S15). ESI-MS (*m*/*z*): 519.0 [M + H]^+^, 541.0 [M + Na]^+^, 557.0 [M + K]^+^.

##### Synthesis of (4-hydroxyphenyl)(3-(4-(4-((7-oxo-7H-furo[3,2-g]chromen-4-yl)oxy)butoxy) phenyl)propyl) diphenylphosphonium iodide (**11**)

(4-Hydroxyphenyl)diphenylphosphine (35.8 mg, 129 µmol, 1.0 eq) and **10** (100 mg, 193 µmol, 1.5 eq) were dissolved in toluene (7.0 mL). The reaction mixture was stirred for 4 days at 110 °C in a sealed vial, then the solvent was removed under reduced pressure. The residue was purified by flash chromatography (DCM/CH_3_OH 95:5 to recover unreacted **10**, then DCM/CH_3_OH 9:1 as eluents) to afford **11** (70.9 mg, 89.0 µmol, 69% yield). ^1^H NMR (400 MHz, CDCl_3_) δ 8.09 (d, *J* = 9.7 Hz, 1H), 7.75–7.67 (m, 2H), 7.64–7.51 (m, 9H), 7.35–7.21 (m, 4H), 7.07–6.97 (m, 3H), 6.97–6.92 (m, 1H), 6.76 (d, *J* = 8.0 Hz, 2H), 6.14 (d, *J* = 9.8 Hz, 1H), 4.50 (t, *J* = 5.6 Hz, 2H), 4.00 (t, *J* = 5.3 Hz, 2H), 3.26–3.14 (m, 2H), 2.81 (t, *J* = 6.7 Hz, 2H), 2.09–1.93 (m, 4H), 1.93–1.80 (m, 2H). ^13^C NMR (101 MHz, CDCl_3_) δ 164.6 (d, *J*_CP_ = 2.4 Hz), 161.4, 158.3, 157.6, 152.6, 149.0, 145.0, 139.6, 135.2 (d, *J*_CP_ = 11.5 Hz), 134.9 (d, *J*_CP_ = 2.3 Hz), 133.3 (d, *J*_CP_ = 9.9 Hz), 131.7, 130.4 (d, *J*_CP_ = 12.4 Hz), 129.9, 119.1 (d, *J*_CP_ = 87.1 Hz), 118.7 (d, *J*_CP_ = 13.2 Hz), 114.7, 113.2, 112.2, 106.5, 105.3, 103.6 (d, *J*_CP_ = 94.7 Hz), 93.6, 72.6, 67.5, 34.9 (d, *J*_CP_ = 16.4 Hz), 26.9, 25.9, 24.5, 22.3 (d, *J*_CP_ = 53.0 Hz) (Figure S16). ESI-MS (*m*/*z*): 669.2 [M-I]^+^.

##### Synthesis of (4-(3-(4-(((1-(*tert*-butoxy)-3-methyl-1-oxopentan-2-yl)carbamoyl)oxy)phenyl) propoxy)phenyl)(3-(4-(4-((7-oxo-7H-furo[3,2-g]chromen-4-yl)oxy)butoxy)phenyl)propyl)diphenylphosphonium 2,2,2-trifluoroacetate (**12**)

We dissolved **11** (1.07 g, 1.34 mmol, 1.0 eq), **4** (659 mg, 1.39 mmol, 1.0 eq) and Et_3_N (374 µL, 2.68 mmol, 2.0 eq) in anhydrous DMF (6.0 mL). The reaction mixture was stirred for 5 h at 80 °C in a sealed vial, then it was diluted with EtOAc (200 mL) and washed with HCl 0.5 M (200 mL). The aqueous layer was extracted with DCM (2 × 150 mL), the combined organic layers were dried over Na_2_SO_4_ and the solvent was removed under reduced pressure. The residue was purified by flash chromatography (DCM/CH_3_OH 97:3 as eluent), then by preparative HPLC (solvent A: H_2_O + 0.1% TFA, solvent B: ACN + 0.1% TFA; %B from 20 to 100 in 35 min) to afford **12** (343 mg, 303 µmol, 23% yield) as a white solid after lyophilization. ^1^H NMR (500 MHz, DMSO) δ 8.13 (d, *J* = 9.8 Hz, 1H), 8.00–7.95 (m, 2H), 7.89–7.82 (m, 2H), 7.77–7.69 (m, 8H), 7.67–7.61 (m, 2H), 7.30–7.23 (m, 4H), 7.21 (d, *J* = 8.5 Hz, 2H), 7.04 (d, *J* = 8.6 Hz, 2H), 6.98 (d, *J* = 8.4 Hz, 2H), 6.79 (d, *J* = 8.6 Hz, 2H), 6.23 (d, *J* = 9.8 Hz, 1H), 4.53 (t, *J* = 5.8 Hz, 2H), 4.09 (t, *J* = 6.2 Hz, 2H), 3.99 (t, *J* = 5.7 Hz, 2H), 3.86 (dd, *J* = 8.0, 6.1 Hz, 1H), 3.50–3.39 (m, 2H), 2.76–2.64 (m, 4H), 2.07–1.98 (m, 2H), 1.98–1.85 (m, 4H), 1.84–1.69 (m, 3H), 1.48–1.32 (m, 10H), 1.29–1.18 (m, 1H), 0.92–0.78 (m, 6H). ^13^C NMR (126 MHz, DMSO) δ 170.8, 163.8 (d, *J*_CP_ = 2.5 Hz), 160.4, 158.5 (q, *J*_CF_ = 34.4 Hz), 157.8, 157.2, 155.0, 152.3, 149.3, 148.9, 146.1, 139.7, 138.1, 135.9 (d, *J*_CP_ = 11.7 Hz), 135.0, 133.5 (d, *J*_CP_ = 10.1 Hz), 132.3, 130.4 (d, *J*_CP_ = 12.4 Hz), 129.5, 129.3, 121.7, 119.3 (d, *J*_CP_ = 86.2 Hz), 116.6 (d, *J*_CP_ = 13.4 Hz), 116.5 (q, *J*_CF_ = 293.6 Hz), 114.6, 113.1, 112.4, 107.9 (d, *J*_CP_ = 92.9 Hz), 106.1, 105.8, 93.3, 81.0, 72.5, 67.7, 67.2, 59.4, 36.5, 34.9 (d, *J*_CP_ = 17.1 Hz), 30.8, 30.2, 27.8, 26.4, 25.5, 25.1, 24.4, 20.6 (d, *J*_CP_ = 51.7 Hz), 15.6, 11.5 (Figure S17). ESI-MS (*m*/*z*): 1016.6 [M-CF_3_COO]^+^.

##### Synthesis of (4-(3-(4-(((1-carboxy-2-methylbutyl)carbamoyl)oxy)phenyl)propoxy)phenyl) (3-(4-(4-((7-oxo-7H-furo[3,2-g]chromen-4-yl)oxy)butoxy)phenyl)propyl)diphenylphosphonium 2,2,2-trifluoroacetate (PAPTPL-I)

We dissolved **12** (238 mg, 211 µmol) in DCM/TFA 1:1 (10 mL) at 0 °C. The reaction mixture was stirred for 2 h at room temperature, then the solvent was removed under reduced pressure. The residue was purified by preparative HPLC (solvent A: H_2_O + 0.1% TFA, solvent B: ACN + 0.1% TFA; %B from 55 to 84 in 13 min) to afford PAPTPL-I (207 mg, 193 µmol, 91% yield) as a white solid after lyophilization. ^1^H NMR (500 MHz, CD_2_Cl_2_) δ 8.13 (d, *J* = 9.8 Hz, 1H), 7.86–7.78 (m, 2H), 7.71–7.63 (m, 4H), 7.61 (d, *J* = 2.3 Hz, 1H), 7.58–7.49 (m, 4H), 7.47–7.39 (m, 2H), 7.15 (d, *J* = 8.2 Hz, 2H), 7.12–6.96 (m, 8H), 6.82 (d, *J* = 8.5 Hz, 2H), 6.16 (d, *J* = 9.8 Hz, 1H), 6.01–5.91 (m, 1H), 4.55 (t, *J* = 5.9 Hz, 2H), 4.26 (br, 1H), 4.08–4.00 (m, 4H), 3.05–2.95 (m, 2H), 2.83–2.71 (m, 4H), 2.17–1.85 (m, 9H), 1.59–1.44 (m, 1H), 1.29–1.13 (m, 1H), 1.02–0.84 (m, 6H). ^13^C NMR (101 MHz, CD_2_Cl_2_) δ 165.1 (d, *J*_CP_ = 2.4 Hz), 161.5, 158.9, 158.3, 155.2, 153.3, 150.1, 149.6, 145.5, 139.9, 138.6, 135.9 (d, *J*_CP_ = 11.6 Hz), 135.8 (d, *J*_CP_ = 2.6 Hz), 133.7 (d, *J*_CP_ = 9.9 Hz), 131.9, 131.0 (d, *J*_CP_ = 12.5 Hz), 130.1, 129.7, 122.2, 119.0 (d, *J*_CP_ = 86.8 Hz), 117.3 (d, *J*_CP_ = 13.7 Hz), 115.2, 113.8, 112.9, 107.2, 107.1 (d, *J*_CP_ = 93.8 Hz), 105.8, 94.0, 73.2, 68.2, 68.0, 38.3, 35.6 (d, *J*_CP_ = 16.5 Hz), 31.7, 30.7, 27.4, 26.5, 25.4, 24.9 (d, *J*_CP_ = 3.4 Hz), 22.6 (d, *J*_CP_ = 53.3 Hz), 15.9, 12.0 (Figure S18). ESI-MS (*m*/*z*): 960.4 [M-CF_3_COO]^+^, 982.3 [M-H-CF_3_COO + Na]^+^.

#### 4.2.3. Peptide Synthesis

Peptides GRKKRRQRRRPPQG (TAT_48–61_) and TFFYGGSRGKRNNFKTEEY (Angiopep-2) were synthesized by standard solid-phase procedures on Wang resin and fluoren-9-ylmethoxycarbonyl (Fmoc) chemistry [70]. Activation employed a 3-fold molar excess of Fmoc-amino acids in DMF solution, HBTU and HOBt, and a 6-fold excess of N,N-diisopropylethylamine (DIPEA) for each coupling cycle. Coupling time was 45 min, room temperature. Fmoc removal was performed with 20% piperidine in DMF. After 5 min of mixing, the solution was replaced for a second 5 min cycle, after which the resin was washed three times with DMF. Since Angiopep-2 contains two consecutive asparagines, which may undergo an α,β-shift upon treatment with piperidine, after their introduction deprotection was carried out instead with a 6% solution of piperazine in DMF [71]. After the Fmoc deprotection of the N-terminus, the peptides linked to the resin were washed three times with DMF, five times with DCM and the residual solvent was removed under reduced pressure.

TAT_48–61_ was synthesized starting with Fmoc-Gly-Wang resin. The side-chain protected amino acids used were Fmoc-Arg(Pbf)-OH, Fmoc-Gln(Trt)-OH, Fmoc-Lys(Boc)-OH.

Angiopep-2 was synthesized starting with Fmoc-Tyr(tBu)-Wang resin. The side-chain protected amino acids used were Fmoc-Glu(OtBu)-OH, Fmoc-Ser(tBu)-OH, Fmoc-Tyr(tBu)-OH, Fmoc-Thr(tBu)-OH, Fmoc-Lys(Boc)-OH, Fmoc-Arg(Pbf)-OH, Fmoc-Asn(Trt)-OH.

#### 4.2.4. Synthesis of TAT-PAPTP

PAPTPL-I (85.6 mg, 79.7 µmol, 1.0 eq), HATU (30.3 mg, 79.7 µmol, 1.0 eq), and DIPEA (27.8 µL, 159 µmol, 2.0 eq) were dissolved in anhydrous DMF (1.0 mL), stirred for 1 h at room temperature and then transferred onto 342 mg of H_2_N-TAT-Wang resin obtained as described in Section 4.2.3. Anhydrous DMF (1.0 mL) was added and the suspension was stirred at room temperature for 19 h. Then it was washed four times with anhydrous DMF and five times with DCM. The residual solvent was removed under reduced pressure. TAT-PAPTP was side-chain deprotected and detached from the resin by treatment with TFA/triisopropyl silane (TIPS) 95:5 for 2 h. At the end of the treatment, TFA was stripped off with N_2_ and the peptide derivative was precipitated by the addition of diethyl ether (Et_2_O). The solid was collected by centrifugation and removal of the supernatant. The residual solvent was removed under reduced pressure. The crude product was dissolved in 0.05% TFA H_2_O/ACN 85:15 *v*/*v* and the solution was filtered on a nylon syringe filter (0.45 μm). TAT-PAPTP was purified by preparative HPLC (solvent A: H_2_O + 0.05% TFA, solvent B: ACN/H_2_O 9:1 + 0.05% TFA; %B 40 for 5 min, from 40 to 65 in 37 min, from 65 to 90 in 1 min, 90 for 1 min). The fractions containing the desired product were collected and lyophilized to constant weight to afford TAT-PAPTP (84.0 mg, 13.8 µmol, 17% yield) (Supplementary Figure S20). ESI-MS (*m*/*z*): 389.2 (calculated 389.1, z = 7), 454.1 (calculated 453.7, z = 6), 544.6 (calculated 544.3, z = 5), 680.4 (calculated 680.1, z = 4), 906.9 (calculated 906.5, z = 3). HRMS (*m*/*z*): 389.0726 (calculated 389.0631, z = 7), 453.7507 (calculated 453.7403, z = 6), 544.2974 (calculated 544.2884, z = 5), 680.1179 (calculated 680.1105, z = 4).

#### 4.2.5. Synthesis of An2-PAPTP

PAPTPL-I (40.0 mg, 37.2 µmol, 1.0 eq), HATU (14.1 mg, 37.2 µmol, 1.0 eq), and DIPEA (13.0 µL, 74.4 µmol, 2.0 eq) were dissolved in anhydrous DMF (1.0 mL), stirred for 1 h at room temperature and then transferred onto 143 mg of H_2_N-Angiopep-2-Wang resin obtained as described in Section 4.2.3. Anhydrous DMF (1.0 mL) was added and the suspension was stirred at room temperature for 21 h. It was successively washed with anhydrous DMF, DCM and then the residual solvent was removed under reduced pressure. An2-PAPTP was side-chain deprotected and detached from the resin by treatment with TFA/TIPS 95:5 for 2 h. At the end of the treatment, TFA was stripped off with N_2_ and the peptide derivative was precipitated by addition of Et_2_O. The solid was collected by centrifugation and removal of the supernatant. Residual solvent was removed under reduced pressure. The crude product was dissolved in 0.05% TFA H_2_O/ACN 1:1 *v*/*v* and then filtered on a nylon syringe filter (0.45 μm). An2-PAPTP was purified by preparative HPLC (solvent A: H_2_O + 0.05% TFA, solvent B: ACN/H_2_O 9:1 + 0.05% TFA; %B 50 for 5 min, from 50 to 70 in 40 min, from 70 to 90 in 1 min, 90 for 1 min). The fractions containing the desired product were collected and lyophilized to constant weight to afford An2-PAPTP (19.1 mg, 4.08 µmol, 11% yield) (Figure S21). ESI-MS (*m*/*z*): 649.6 (calculated 649.3, z = 5), 811.8 (calculated 811.4, z = 4), 1081.9 (calculated 1081.5, z = 3). HRMS (*m*/*z*): 649.3058 (calculated 649.2933, z = 5), 811.3754 (calculated 811.3667, z = 4), 1081.5011 (calculated 1081.4889, z = 3).

### 4.3. Synthesis of PAPTPL

(4-(3-(4-hydroxyphenyl)propoxy)phenyl)(3-(4-(4-((7-oxo-7H-furo[3,2-g]chromen-4-yl)oxy)butoxy)phenyl)propyl)diphenylphosphonium 2,2,2-trifluoroacetate (PAPTPL) was obtained as side product of the reaction to produce **12** from **11** and **4**. It was purified from the reaction mixture by preparative HPLC (solvent A: H_2_O + 0.1% TFA, solvent B: ACN + 0.1% TFA; %B from 20 to 100 in 35 min) and obtained as a white solid after lyophilization (Supplementary Figure S22). ^1^H NMR (500 MHz, DMSO) δ 8.14 (d, *J* = 9.8 Hz, 1H), 7.97 (d, *J* = 2.3 Hz, 1H), 7.89–7.82 (m, 2H), 7.77–7.69 (m, 8H), 7.68–7.60 (m, 2H), 7.30–7.22 (m, 4H), 7.03 (d, *J* = 8.6 Hz, 2H), 6.99 (d, *J* = 8.4 Hz, 2H), 6.79 (d, *J* = 8.6 Hz, 2H), 6.66 (d, *J* = 8.4 Hz, 2H), 6.24 (d, *J* = 9.8 Hz, 1H), 4.54 (t, *J* = 5.8 Hz, 2H), 4.06 (t, *J* = 6.3 Hz, 2H), 4.00 (t, *J* = 5.8 Hz, 2H), 3.50–3.40 (m, 2H), 2.68 (t, *J* = 7.5 Hz, 2H), 2.61 (t, *J* = 7.6 Hz, 2H), 2.02–1.86 (m, 6H), 1.81–1.69 (m, 2H). ^13^C NMR (126 MHz, DMSO) δ 163.8 (d, *J*_CP_ = 2.3 Hz), 160.4, 157.8, 157.2, 155.6, 152.3, 148.9, 146.1, 139.7, 135.9 (d, *J*_CP_ = 11.6 Hz), 135.0, 133.5 (d, *J*_CP_ = 10.1 Hz), 132.3, 131.3, 130.4 (d, *J*_CP_ = 12.4 Hz), 129.5, 129.4, 119.3 (d, *J*_CP_ = 86.2 Hz), 116.6 (d, *J*_CP_ = 13.5 Hz), 115.3, 114.6, 113.1, 112.4, 107.9 (d, *J*_CP_ = 93.0 Hz), 106.1, 105.8, 93.3, 72.5, 67.7, 67.2, 34.9 (d, *J*_CP_ = 16.7 Hz), 30.6, 30.5, 26.4, 25.5, 24.4 (d, *J*_CP_ = 3.6 Hz), 20.5 (d, *J*_CP_ = 51.3 Hz) (Figure S19). ESI-MS (*m*/*z*): 803.4 [M-CF_3_COO]^+^.

### 4.4. Solubility Determinations

For TAT-PAPTP and An2-PAPTP, three distinct suspensions were prepared by diluting 25 mM stock solutions in DMSO to 100 μM, in 0.9% NaCl saline or water (DMSO < 0.4%). The suspensions were sonicated 5 min and immediately analyzed. The suspensions were then centrifuged at 12,000× *g*, 18 °C, for 1 and 2 h, and the supernatants were analyzed by HPLC/UV at the end of each centrifugation. An additional analysis was performed after 24 h of standing. The chromatographic peak areas were used to determine the concentration of the compound in the supernatants, based on calibration curves built using solutions in DMSO.

### 4.5. Cell Cultures

SHSY-5Y cells (kindly provided by I. Szabò) were grown in Dulbecco’s Modified Eagle Medium (DMEM, Aurogene) supplemented with 10% (*v*/*v*) fetal bovine serum (FBS), 100 U/mL penicillin G, 0.1 mg/mL streptomycin and 5% glutamine.

### 4.6. MTS Assay

SHSY-5Y cells were seeded (10,000 cells/well) in standard 96-well plates in DMEM (200 µL). After 24 h, the growth medium was replaced with a medium containing PAPTP and PAPTPL (from a stock solution in DMSO) at the desired final concentrations (20, 10 and 3 μM). DMEM without additions (CONTROL in Figure 2) was used as negative control. The final concentration of DMSO was 0.1% or lower in all cases (including control). After incubation for 24 h, 20 µL of CellTiter 96 AQUEOUS One solution (Promega, Italy) were added to each well and the plate was incubated at 37 °C in a humidified, 5% CO_2_ atmosphere for 4 h. Absorbance was measured at 490 nm to detect formazan formation.

### 4.7. Quantification of PAPTPL and Peptide-PAPTP Conjugates in Tissues

Different analytical protocols were set-up to calculate the recovery yields of TAT-PAPTP and An2-PAPTP from tissues.

#### 4.7.1. Recovery of PAPTPL

The liver, spleen, kidneys, heart obtained from untreated mice were weighed and homogenized with PBS pH 7.4 (1 vol) with a potter. The pre-established amount of PAPTPL was added into tissue homogenates or blood and, after thorough vortexing, 5 vol of acetone were added and the sample was sonicated for 2 min. Subsequently, 5 vol of ACN containing 0.1% TFA were added and the sample was sonicated for 5 min. The sample was then centrifuged at 12,000× *g* for 7 min at 4 °C and the supernatant was collected, concentrated with a Hetovac VR-1, and analyzed via HPLC-UV.

The concentration of the analyte in the sample was calculated from the calibration curve, where A_312 nm_ = 6.33 × Concentration (µM).

The volume of the sample analyzed was carefully measured to calculate the amount of analyte: µmol = concentration (μM)*Volume (L).

The recovery yields were calculated as follows: (Total µmol quantified via HPLC/µmol spiked) × 100.

The recovery yields for liver, spleen, kidneys, heart and blood were 82 ± 15%, 99 ± 5%, 55 ± 1%, 57 ± 6% and 80 ± 8%, respectively.

#### 4.7.2. Recovery of TAT-PAPTP from Brain

The protocol set up for PAPTPL recovery turned out to be inefficient for the quantification of TAT-PAPTP. An alternative protocol was thus developed. Tissue obtained from untreated mice was weighed and homogenized with PBS pH 7.4 (1 vol) with a potter. The pre-established amount of analyte was added into tissue homogenates and, after thorough vortexing, 1 vol of PBS pH 12.5 was added and the sample was incubated with intermittent vortexing at 60 °C for 24 h. This step is required to allow the hydrolysis of the carbamate bond connecting the peptide and PAPTPL. 10 vol of ethyl acetate were added and, after vortexing and sonication for 2 min, the sample was centrifuged at 12,000× *g*, 7 min, Room Temperature. The organic phase was collected and concentrated with Hetovac VR-1. ACN was added to the concentrated sample to allow the resuspension of PAPTPL and analyzed via HPLC. Quantification was as described for PAPTPL.

#### 4.7.3. Recovery of An2-PAPTP

Brain and liver obtained from untreated mice were weighed and homogenized with PBS pH 7.4 (1 vol) with a potter. The pre-established amount of analyte was added into tissue homogenates and, after thorough vortexing, 5 vol of acetone were added and the sample was sonicated for 2 min. Subsequently, 5 vol of ACN containing 0.1% trifluoracetic acid (TFA) were added and the sample was sonicated for 5 min. The sample was then centrifuged at 12,000× *g* for 7 min at 4 °C and the supernatant was collected, concentrated with a Hetovac VR-1, and analyzed via HPLC-UV.

This protocol turned out to suitable for quantification of PAPTPL released by hydrolysis in the tissue but was inefficient for the quantification of An2-PAPTP. Only a very small fraction—depending on tissue—of the spiked quantity could be recovered. The most likely reason may reside in the intrinsic ability of positively charged peptides to attach to biological components, e.g., DNA or phospholipids, avoiding extraction into the organic solvents and remaining thus “trapped” in the pellet.

**Scheme 3:**
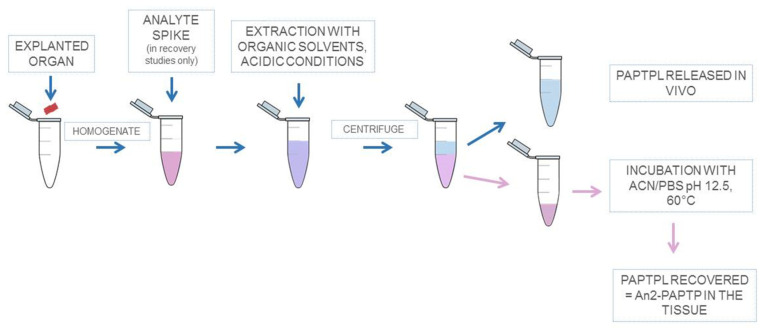
Graphical representation of the protocol adopted for the extraction of An2-PAPTP from brain and liver.

To quantify the amount of An2-PAPTP stuck in the tissue, the pellet remaining after the procedure described above was incubated with intermittent Vortexing at 60 °C for 3 h with PBS pH 12.5/ACN 1:1 and then centrifuged at 12,000× *g* for 7 min. HPLC-UV analysis of the supernatant, measuring PAPTPL hydrolyzed from the tissue, thus indirectly reflects the amount of An2-PAPTPL in the pellet (Scheme 3). Quantification was as described for PAPTPL.

The recovery yields from brain and liver were 58 ± 11% and 60 ± 12%, respectively. A scheme of the analytical procedure is depicted below.

### 4.8. Stability in Blood

C57BL mice were anesthetized and blood was withdrawn from the jugular vein and heparinized. Blood samples (1 mL) were spiked with compound (5 µM; dilution from a 5 mM stock solution in DMSO) and incubated at 37 °C for 4 h (the maximum period allowed by blood stability). Aliquots were taken after 10 min, 30 min, 1 h, 2 h, and 4 h before HPLC-UV analysis.

Each aliquot was treated as described above (Section 4.7.1). Compound stability in blood was expressed as % of PAPTPL released relative to the amount of An2-PAPTP and TAT-PAPTP spiked in the sample at time 0.

### 4.9. In Vivo Experiments

C57BL/6 mice were housed in the facility of the Department of Biology (Padova); food and water were provided ad libitum. All procedures were approved by the University of Padova Ethical Committee for Animal Welfare (OPBA) and by the Italian Ministry of Health (Permit Number 111/2017-PR), and conducted with the supervision of the Central Veterinary Service of the University of Padova, in compliance with Italian Law DL 26/2014, embodying UE Directive 2010/63/EU.

A stock solution of the peptide-PAPTP conjugates was prepared in DMSO. TAT-PAPTP was administered at 8 and 5 μmol/kg bw and An2-PAPTP only at 5 μmol/kg bw. The calculated quantity of drugs was diluted from the stock solution in saline or mouse plasma to a final volume of 200 μL.

### 4.10. Planar Bilayer Experiments with An2-PAPTP and TAT-PAPTP

The possible effects of An2-PAPTP and TAT-PAPTP on phospholipid membranes were investigated using the lipid bilayer technique. Bilayers with a capacity of 100–150 pF were prepared “painting” a diphytanoylphosphatidylcholine (DPhPC, Avanti Polar Lipids) solution in octane across a 150-µm hole in a Delrin cuvette (Warner Instruments, a division of Harvard Bioscience, Inc., Holliston, MA, USA). The two compartments separated by the bilayer are identified as cis and trans, and all voltages refer to the cis side, zero being assigned to the trans (grounded) one. One mL of recording solution (150 mM KCl, 10 mM HEPES/KOH, pH 7.4) was placed in both compartments. The contents of both chambers were stirred by magnetic bars when necessary. Lack of activity in the membrane before the addition of a compound was monitored for at least 5 min in each experiment, and long-lasting (≥15 min) control experiments (no addition) showed no activity. An2-PAPTP or TAT-PAPTP (DMSO stock solution) in a final concentration of 0.5 µM were added to the cis side. The final concentration of DMSO was 0.6%. 2 µM gramicidin from Bacillus brevis (Sigma) was added to the cis side as control.

Electrical connections were made using Ag/AgCl electrodes and agar salt bridges to minimize eliminate liquid junction potentials. Data were acquired at 10 kHz using a Bilayer Clamp BC-525C amplifier (Warner Instruments, a division of Harvard Bioscience, Inc., Holliston, MA, USA.) and low-pass filtered at 300 Hz for presentation. Digitization was performed by a Digidata 1322 A interface and pClamp software (Molecular Devices, pCLAMP_10).

### 4.11. Haemolysis Assays

Haemolysis studies were conducted as described in [72]. Briefly, freshly withdrawn blood samples were centrifuged at 1000× *g* for 10 min to obtain erythrocytes; the pellet was then washed thrice with sterile PBS (pH 7.4). Erythrocytes (5 × 10^7^ cells in 1 mL of PBS for each experimental condition) were incubated with 2, 1 or 0.5 µM An2-PAPTP or TAT-PAPTP for 15 min. The samples were then centrifuged and absorbance (Abs) of the supernatant was measured at 540 nm. Triton X-100 1% and PBS were used as positive and negative control, respectively.

The percentage of haemolysis was calculated with the following equation:

% haemolysis = [(Abs_sample_ − Abs_PBS_)/Abs_Triton X-100_ − Abs_PBS_)] × 100

### 4.12. Statistics

Significance in comparisons was assessed using one-way analysis of variance (ANOVA), followed by Tukey’s multiple comparisons test.

## 5. Conclusions

Targeting and inhibiting the mitochondrial Kv1.3 channel is emerging as a novel and promising strategy to selectively kill cancer cells. Whilst the mitochondria-targeted Kv1.3 inhibitors PCARBTP and PAPTP have proven their efficacy and lack of side effects in in vivo models of melanoma and pancreatic adenocarcinoma, BBB hinders their action against brain tumors.

In this proof-of-concept work, we showed that conjugation of PAPTP with cell- or brain-penetrating peptides may represent a successful strategy to allow brain delivery of PAPTP.

Furthermore, the chemical strategy described here offers the possibility to conjugate PAPTP to small peptides allowing tumor-specific accumulation of the drug, an approach currently being investigated in our labs.

## Figures and Tables

**Table 1:** Brain levels of PAPTPL in An2-PAPTP treated mice. Quantifications refer to the amount of PAPTPL extracted from brains after hydrolysis of the carbamate bond, corresponding to the amount of An2-PAPTP or its fragments. Data in the table are expressed as mean ± SEM (*n* = 5 and 4, respectively).

Time of Treatment (min)	Psoralenic Species (nmol/g Tissue)	Psoralenic Species (% of Injected Dose)
15	0.3 ± 0.1	0.10 ± 0.04
30	0.4 ± 0.6	0.2 ± 0.2

**Table 2:** Quantification of An2-PAPTP-derived metabolites in the liver. Data are expressed as mean ± SEM (*n* = 5, 4, and 6 for 15, 30 and 60 min, respectively).

Time of Treatment (min)	PAPTPL	PAPTPL-I	PAPTPL-IT	PAPTPL-ITF
	**nmol/g fresh tissue**			
15	1.5 ± 0.6	0.5 ± 0.3	3.6 ± 1.3	2.2 ± 0.6
30	1.1 ± 0.3	0.2 in one animal out of four	3.6 ± 2.6	1.7 ± 0.6
60	0.4 ± 0.2	5.4 ± 1.6	7.5 ± 2.1	4.5 ± 1.0
	**% administered dose**			
15	1.2 ± 0.5	0.4 ± 0.2	2.8 ± 1.0	1.7 ± 1.0
30	0.9 ± 0.2	0.03 in one animal out of four	2.7 ± 1.8	1.2 ± 0.5
60	0.4 ± 0.1	5.0 ± 1.8	6.2 ± 1.6	3.9 ± 1.0

**Table 3:** Quantification of all the species with a psoralenic ultraviolet (UV) absorption spectrum extracted from the liver, and detected at 312 nm (assuming the same molar extinction coefficient for all species). No correction for recovery yield was applied. Data in the table are expressed as mean ± SEM (*n* = 5, 4, and 6 for 15, 30 and 60 min, respectively).

Time of Treatment (min)	Psoralenic Species (nmol/g Tissue)	Psoralenic Species (% of Injected Dose)
15	23 ± 3	20 ± 3
30	17 ± 2	16 ± 1
60	11 ± 3	12 ± 1

**Table 4:** Quantification of all the species with a psoralenic UV absorption spectrum extracted from the spleen, kidneys or heart, and detected at 312 nm (assuming the same molar extinction coefficient for all species). No correction for recovery yield was applied. Data in the table are expressed as mean ± SEM (*n* = 5, 4, and 6 for 15, 30 and 60 min, respectively).

Time of Treatment (min)	Psoralenic Species (nmol/g Tissue)	Psoralenic Species (% of Administered Dose)
**SPLEEN**		
15	28 ± 9	1.2 ± 0.3
30	4.3 ± 1.4	0.27 ± 0.09
60	11 ± 3	0.8 ± 0.2
**KIDNEY**		
15	5 ± 4	1.2 ± 0.8
30	1.3 ± 0.2	0.32 ± 0.07
60	1.2 ± 0.1	0.31 ± 0.03
**HEART**		
15	0.2 ± 0.1	0.07 ± 0.01
30	0.36 in one animal out of four	0.07 in one animal out of four
60	0	0

## Supplementary Materials

The following are available online at https://www.mdpi.com/1424-8247/14/2/129/s1.

## Abbreviations

An2	Angiopep2
ACN	acetonitrile
AUC	area under the curve
BBB	blood-brain barrier
bw	body weight
CD	circular dichroism
CLL	chronic lymphocytic leukemia
CNS	central nervous system
CPP	cell-penetrating peptide
DCM	dichloromethane
DIPEA	N,N-diisopropylethylamine
DMAP	4-dimethylaminopyridine
DMEM	Dulbecco’s Modified Eagle Medium
DMF	N,N-dimethylformamide
DMSO	dimethylsulfoxide
DOX	doxorubicin
DTX	docetaxel
ESI	electrospray ionization
EtOAc	ethyl acetate
Et2O	diethyl ether
Fmoc	fluoren-9-ylmethoxycarbonyl
HATU	1-[bis(dimethylamino)methylene]-1H-1,2,3-triazolo[4,5-b]pyridinium 3-oxide hexafluorophosphate
HBTU	2-(1H-benzotriazol-1-yl)-1,1,3,3-tetramethyluronium hexafluorophosphate
HOBt	1-hydroxybenzotriazole
HPLC	high-performance liquid chromatography
HRMS	high-resolution mass spectrometry
IMM	inner mitochondrial membrane
i.p.	intraperitoneal
i.v.	intravenous
Kv1.3	voltage-dependent potassium channel 1.3
LRP-1	low-density lipoprotein receptor related Protein-1
MDR	multiple drug resistance
MS	mass spectrometry
MTS	3-(4,5-dimethylthiazol-2-yl)-5-(3-carboxymethoxyphenyl)-2-(4-sulfophenyl)-2H-tetrazolium
PBS	phosphate-buffered saline
PDAC	pancreatic ductal adenocarcinoma
PTX	paclitaxel
r.t.	room temperature
SEM	standard error of the mean
TEA	triethylamine
TFA	trifluoroacetic acid
TIPS	triisopropyl silane
TPP	triphenylphosphonium
WT	wild type
