# Supplementary material for: An Angiopep2-PAPTP Construct Overcomes the Blood-Brain Barrier. New Perspectives against Brain Tumors
Source: Pharmaceuticals (Basel). 2021 Feb 6;14(2):129. doi: 10.3390/ph14020129 (PMC7914648; doi:10.3390/ph14020129)

SUPPLEMENTARY MATERIAL

An Angiopep2-PAPTP construct overcomes the blood-brain-barrier. New perspectives against brain tumors.

Sofia Parrasia [1] †, Andrea Rossa[2] †, Tatiana Varanita[3], Vanessa Checchetto[3], Riccardo De Lorenzi[2,4], Mario Zoratti[1,5], Cristina Paradisi[2], Paolo Ruzza[2,4], Andrea Mattarei[6], Ildikò Szabò[3], Lucia Biasutto[1,5,*]

[1] Dept. Biomedical Sciences, University of Padova, Viale G. Colombo 3, 35131 Padova, Italy
[2] Dept. Chemical Sciences, University of Padova, Via F. Marzolo 1, 35131 Padova, Italy
[3] Dept. Biology, University of Padova, Viale G. Colombo 3, 35131 Padova, Italy
[4] CNR Institute of Biomolecular Chemistry, Via F. Marzolo 1, 35131 Padova, Italy
[5] CNR Neuroscience Institute, Viale G. Colombo 3, 35131 Padova, Italy
[6] Dept. Pharmaceutical and Pharmacological Sciences, University of Padova, Via F. Marzolo 5, 35131 Padova, Italy

† These authors contributed equally to this work and thus share first authorship
* Correspondence: lucia.biasutto@cnr.it

Content:

Structures of Model 1, Model 2, PCARBTP and PAPTPOH p. 2

CD characterization of TAT and TAT-PAPTP p. 3-4

CD characterization of Angiopep-2 and An2-PAPTP p. 5-6

Material and Methods, References and abbreviations for CD characterization p. 7-8

Quantification of An2-PAPTP-derived metabolites in spleen, kidneys and heart p. 9-11

^{1}H and ^{13}C NMR spectra of compounds **1-12**, PAPTPL-I and PAPTPL p. 12-25

HPLC-UV chromatograms of TAT-PAPTP, An2-PAPTP and PAPTPL p. 26-27

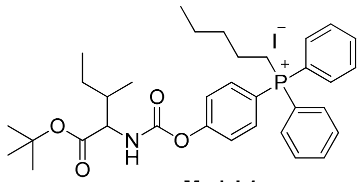

Model 1

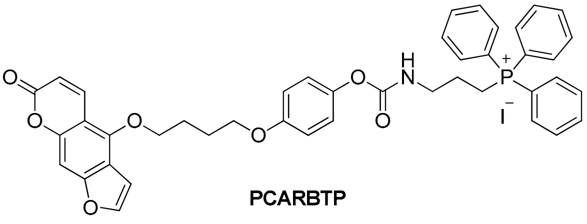

PCARBTP

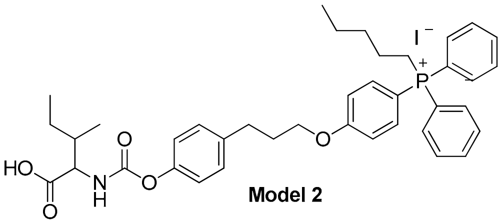

Model 2

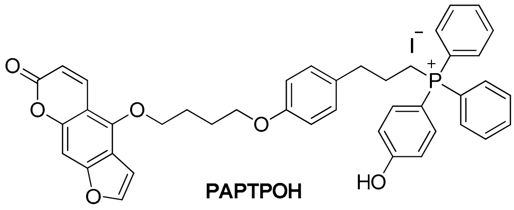

PAPTPOH

Supplementary Figure S1. *Structures of Model 1, Model 2, PCARBTP and PAPTPOH.*

CD characterization of TAT and TAT-PAPTP

The conformation properties of free and labelled TAT peptide were evaluated in aqueous and membrane-mimicking environments by Circular Dychroism (CD) spectroscopy. In previous work, we demonstrated that the conformation adopted by TAT depends on temperature: at low temperature (10°C) a PolyProline II (PPII)-helix characterized by three residues per turn predominates [1]. Residues at position *i* and *i*+3 lie on the same edge of the helix. Thus, positively charged residues are located on different edges of the helix minimizing the repulsion energy between the positively charged side-chains [2]. This conformation is present also at room temperature in phosphate buffer and is characterized by a strong negative band at about 199 nm and a weak positive band in the 220-230 nm range. Raising the temperature, the positive band disappeared, and at 40°C, the CD spectrum became negative over the entire wavelength range with in addition a decrease in the intensity of the negative band, suggesting the presence of a random coil conformation.

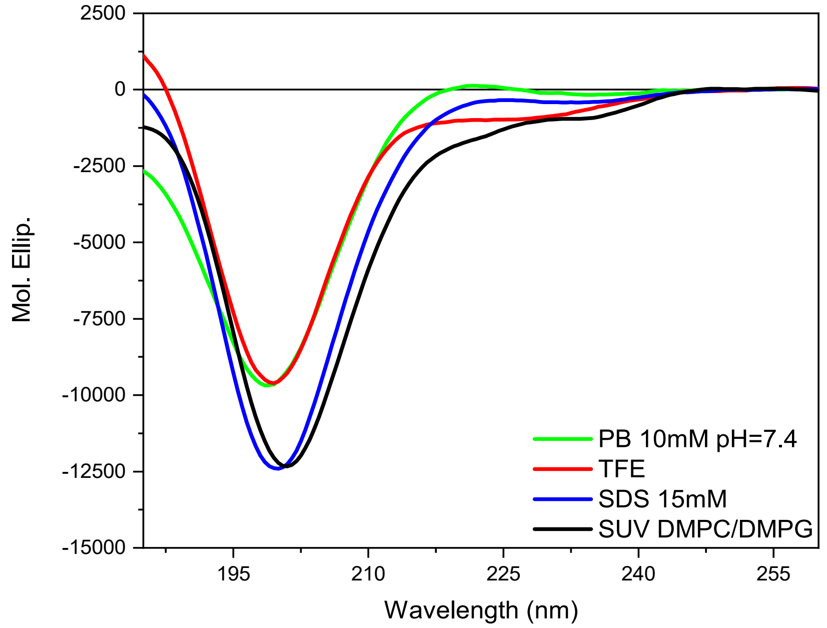

Supplementary Figure S2. *Far-UV CD spectra of TAT_{48-61} peptide in: 10 mM PB, pH 7.4 (green line); 8:2 v/v TFE/H_2O (red line); 15 mM SDS micelles (blue line); DMPC/DMPG 3:1 SUVs (black line). Peptide concentration was 0.2 mg/mL. The spectra show the average of 9 recordings.*

The inability of membrane-mimicking environments, namely micellar Sodium Dodecyl Sulphate (SDS), a TriFluoro Ethanol (TFE)/water mixture and 1,2-DiMyristoyl-sn-glycero-3-PhosphoCholine - 1,2-DiMyristoyl-sn-glycero-3-PhosphorylGlycerol (DMPC-DMPG) (3:1 w/w) Small Unilamellar Vesicles (SUVs), to induce an ordered α-helix or β-sheet structure in unlabelled TAT peptide supports the view that this peptide is folded in a stable PPII helix conformation even in this environment.

The conjugation of PAPTP to the N-terminal amino group of the TAT peptide only partially changed the CD spectra, suggesting that the cargo did not influence the structure of the peptide and that the PPII helix conformation is favourite also in the presence of the cargo.

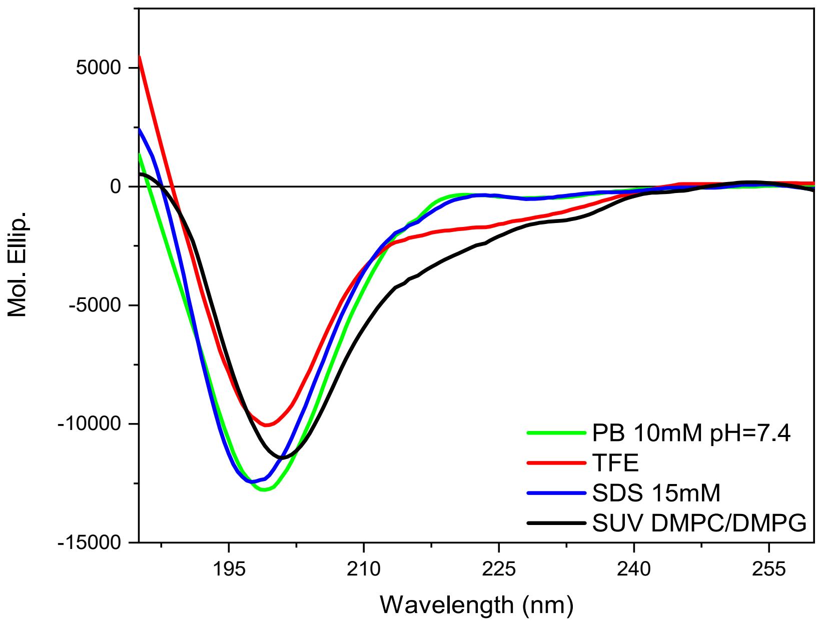

Supplementary Figure S3. *Far-UV CD spectra of TAT-PAPTP in: 10 mM PB, pH 7.4 (green line); 8:2 v/v TFE/H_2O (red line); 15 mM SDS micelles (blue line); DMPC/DMPG 3:1 SUVs (black line). Peptide concentration was 0.2 mg/mL. The spectra show the average of 9 recordings.*

CD characterization of Angiopep-2 and An2-PAPTP

The CD spectrum of Angiopep2 in aqueous solution is characterized by the presence of two low-intensity bands: a negative band at 203 nm and a positive band at about 220 nm. This spectrum was described by Perczel *et al.* as characteristic of a "not-typical" secondary structure [3]. In TFE/water solution, the positive band disappeared and the negative band was shifted to longer wavelengths (205 nm), while a new negative band centred at 230 nm appeared. A strong negative band at about 194 nm appeared in the presence of either SDS micelles or DMPC-DMPG SUVs. The positive band observed in aqueous solution was preserved in micellar SDS solution, but was absent in the presence of SUVs, suggesting different types of interaction of the peptide with micelles and vesicles.

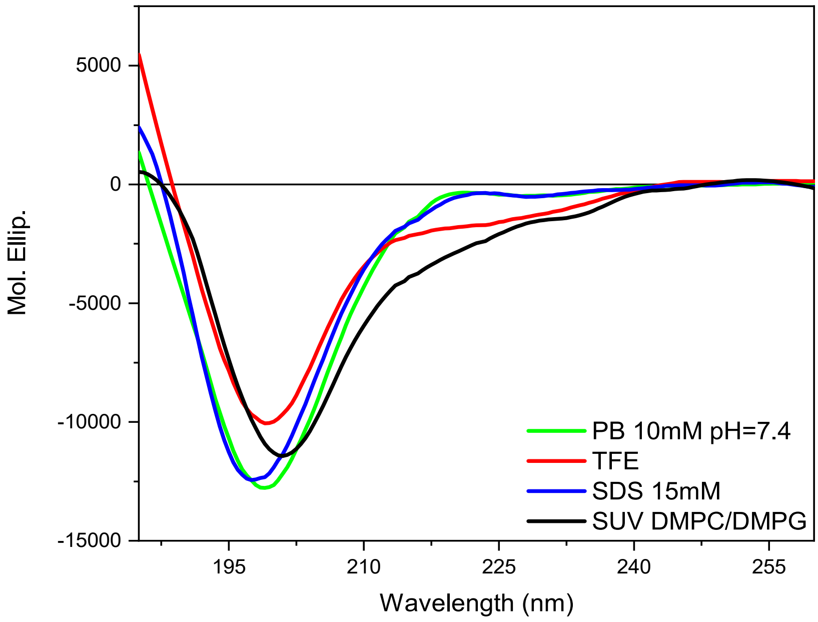

Supplementary Figure S4. *Far-UV CD spectra of Angiopep-2 in: 10 mM PB, pH 7.4 (green line); 8:2 v/v TFE/H2O (red line); 15 mM SDS micelles (blue line); DMPC/DMPG 3:1 SUVs (black line). Peptide concentration was 0.2 mg/mL. The spectra show the average of 9 recordings.*

Surprisingly, the secondary structure of Angiopep2 appears to be profoundly modified by the conjugation of the PAPTP cargo. The CD spectrum could not be recorded in Phosphate Buffer (PB) solution, while in Tris-HCl buffer only a low-intensity signal was produced, suggesting a negative effect of the phosphate buffer on the solubility of Angiopep2-PAPTP, probably due to an

interaction of the cargo molecule with phosphate ions. This effect was not observed in the TAT construct, presumably due to the high hydrophilicity of the cell-penetrating peptide.

The CD spectrum of An2-PAPTP recorded in TFE solution was characterized by a strong positive band at about 193 nm and a negative band at 207 nm with a shoulder at 222 nm indicating the presence of an ordered conformation. The secondary structure estimation analysis performed using the CDApps software [4] revealed the presence of high content of β-strand structure (38%).

On the other hand, CD spectra both in micellar SDS solution and in the presence of DMPC/DMPG SUVs are characterized by a strong negative band at short wavelengths, not attributable to any canonical secondary structure.

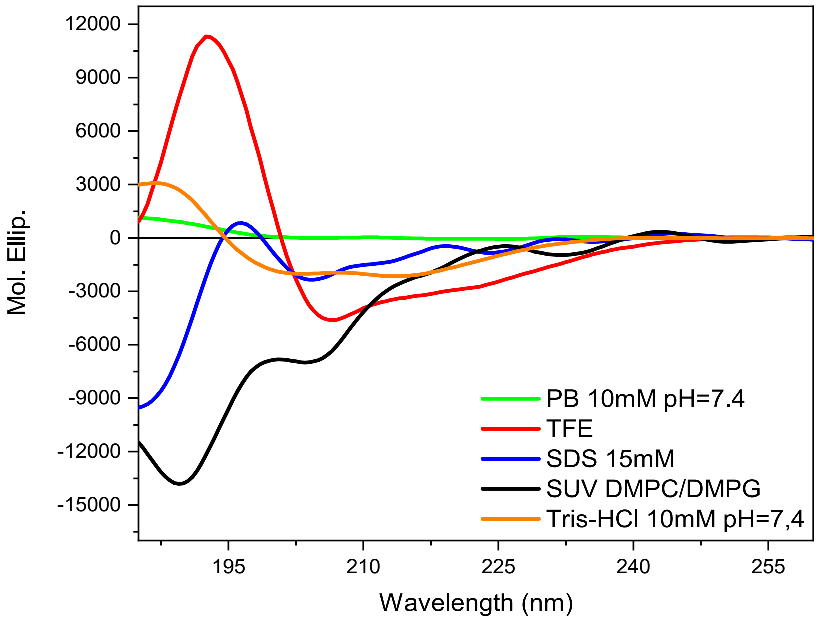

Supplementary Figure S5. *Far-UV CD spectra of An2-PAPTP in: 10 mM PB, pH 7.4 (green line); 8:2 v/v TFE/H2O (red line); 15 mM SDS micelles (blue line); DMPC/DMPG 3:1 SUVs (black line); 10 mM Tris-HCl buffer, pH 7.4. Peptide concentration was 0.2 mg/mL. The spectra show the average of 9 recordings.*

Materials and Methods

Peptide/peptide conjugate samples were dissolved in deionized water at the concentration of 1 mg/mL and then diluted with the desired solutions to a final concentration of 0.2 mg/mL. The concentration of the buffer stock solutions was increased by 25% to compensate for the dilution upon the addition of the stock peptide solutions. Far-UV CD curves were recorded with a JASCO J-1500 spectropolarimeter, at 25 ± 0.1 °C. The CD data were obtained as the mean of 9 scans, at a rate of 50 nm/min, with data pitch of 0.5 nm, response time of 2 s and 1 nm bandwidth. The buffer contribution was subtracted.

Liposome preparation for CD studies. Liposomes were obtained by the dissolution of 22.56 mg (3 mmol) of DMPC and 7.44 mg (1 mmol) of DMPG in 1 mL of tert-butanol. The solution was lyophilized and then suspended in 3 mL of phosphate buffer 10 mM pH 7.4. The resulting suspension was then sonicated with a Cole-Parmer Ultrasonic Processor GEX 400 (Vernon Hills, IL, USA) for 30 minutes (cycle: 3.5 seconds on and 1 second off), at 20% amplitude and filtered with a 0.05 μm filter to retain only liposomes smaller than 0.05 μm [5].

References

[1] Ruzza, P., Calderan, A., Guiotto, A., Osler, A., Borin, G. Tat cell-penetrating peptide has the characteristics of a poly(proline) II helix in aqueous solution and in SDS micelles. *J. Pept. Sci.* **2004**, *10*, 423-426.

[2] Krimm, S., Mark, J. E. Conformations of polypeptides with ionized side chains of equal length. *Proc. Natl. Acad. Sci. USA* **1968**, *60*, 1122-1129.

[3] Perczel, A., Hollosi, M., Sandor, P., Fasman, G. D. The evaluation of type I and type II β-turn mixtures. *Int. J. Pept. Protein Res.* **1993**, *41*, 223-236.

[4] Hussain, R., Benning, K., Javorfi, T., Longo, E., Rudd, T. R., Pulford, B., Siligardi, G. CDApps: integrated software for experimental planning and data processing at beamline B23, Diamond Light Source. *J. Synchrotron Radiat.* **2015**, *22*, 465-468.

[5] Ruzza, P., Donella-Deana, A., Calderan, A., Brunati, A., Massimino, M. L., Elardo, S., Mattiazzo, A., Pinna, L. A., Borin, G. Antennapedia/HS1 chimeric phosphotyrosyl peptide: conformational properties, binding capability to c-Fgr SH2 domain and cell permeability. *Biopolymers* **2001**, *60*, 290-306.

Abbreviations

CD: Circular Dichroism; PB: Phosphate Buffer; TFE: trifluoroethanol; DMPC: 1,2-dimyristoyl-sn-glycero-3-phosphocholine; DMPG: 1,2-dimyristoyl-sn-glycero-3-phosphorylglycerol; SUV: Small Unilamellar Vesicle

SPLEEN				
**Time of treatment (min)**	PAPTPL	PAPTPL-I	PAPTPL-IT	PAPTPL-ITF
		**nmol/g fresh tissue**		
**15**	0.9 ± 0.2	0.7 ± 0.3	1.9 ± 0.7	2 ± 1
**30**	0.9 ± 0.3	0	1.2 ± 0.7	0.3 ± 0.2
**60**	1.3 ± 0.2	1.2 ± 0.4	1.2 ± 0.5	1.2 ± 0.2
		**% administered dose**		
**15**	0.05 ± 0.02	0.04 ± 0.02	0.10 ± 0.04	0.13 ± 0.08
**30**	0.06 ± 0.02	0	0.07 ± 0.04	0.02 ± 0.01
**60**	0.10 ± 0.02	0.08 ± 0.03	0.07 ± 0.03	0.08 ± 0.01

Supplementary Table S1. *Quantification of An2-PAPTP-derived metabolites in the spleen. Data are expressed as mean ± SEM (N = 5, 4, and 6 for 15, 30 and 60 minutes, respectively).*

KIDNEYS				
**Time of treatment (min)**	PAPTPL	PAPTPL-I	PAPTPL-IT	PAPTPL-ITF
**nmol/g fresh tissue**				
**15**	0.7 ± 0.1	0.32 in one animal out of five	0.72 in one animal out of five	0.15 in one animal out of five
**30**	0.7 ± 0.2	0.32 in one animal out of four	0.55 in one animal out of four	0.4 ± 0.1
**60**	1.1 ± 0.2	0	0.07 in one animal out of six	0.2 ± 0.1
**% administered dose**				
**15**	0.1 ± 0.1	0.02 in one animal out of five	0.04 in one animal out of five	0.01 in one animal out of five
**30**	0.03 ± 0.02	0.03 in one animal out of four	0.05 in one animal out of four	0.12 ± 0.08
**60**	0.09 ± 0.02	0	0.006 in one animal out of six	0.02 ± 0.01

Supplementary Table S2. *Quantification of An2-PAPTP-derived metabolites in the kidneys. Data are expressed as mean ± SEM (N = 5, 4, and 6 for 15, 30 and 60 minutes, respectively).*

HEART				
**Time of treatment (min)**	PAPTPL	PAPTPL-I	PAPTPL-IT	PAPTPL-ITF
**nmol/g fresh tissue**				
**15**	0.12 ± 0.06	0	0	0
**30**	0.1 ± 0.1	0	0.10 ± 0.08	0.08 ± 0.07
**60**	0.19 ± 0.05	0	0	0
**% administered dose**				
**15**	0.013 ± 0.007	0	0	0
**30**	0.01 ± 0.01	0	0.01 ± 0.01	0.008 ± 0.007
**60**	0.018 ± 0.005	0	0	0

Supplementary Table S3. *Quantification of An2-PAPTP-derived metabolites in the heart. Data are expressed as mean ± SEM (N = 5, 4, and 6 for 15, 30 and 60 minutes, respectively).*

Supplementary Figure S6. *4-(3-chloropropyl)phenol (**1**)*: ^{1}H-NMR (400 MHz, CDCl3) and ^{13}C-NMR (101 MHz, CDCl3)

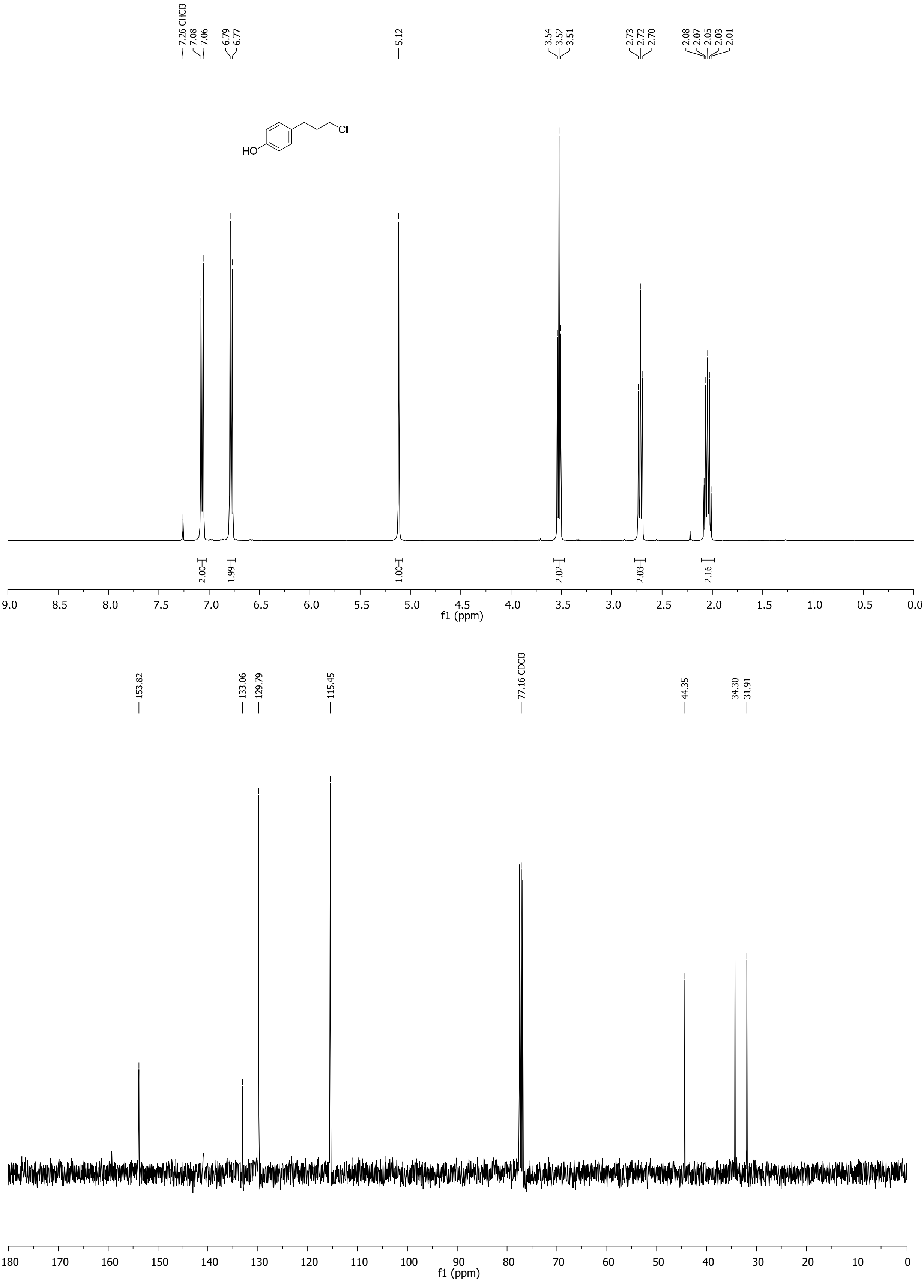

Supplementary Figure S7. *tert-butyl 3-methyl-2-(((4-nitrophenoxy)carbonyl)amino)pentanoate (**2**)*: ^{1}H-NMR (500 MHz, CDCl3) and ^{13}C-NMR (126 MHz, CDCl3)

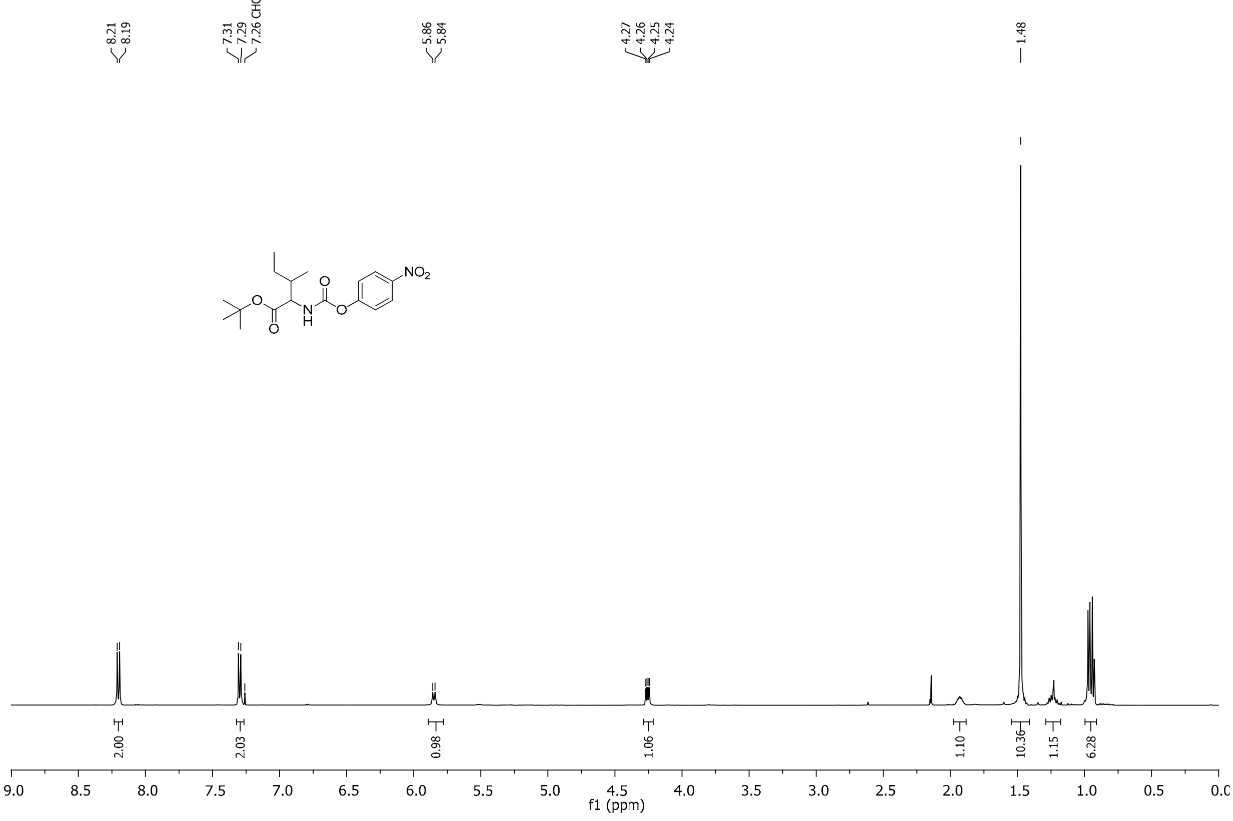

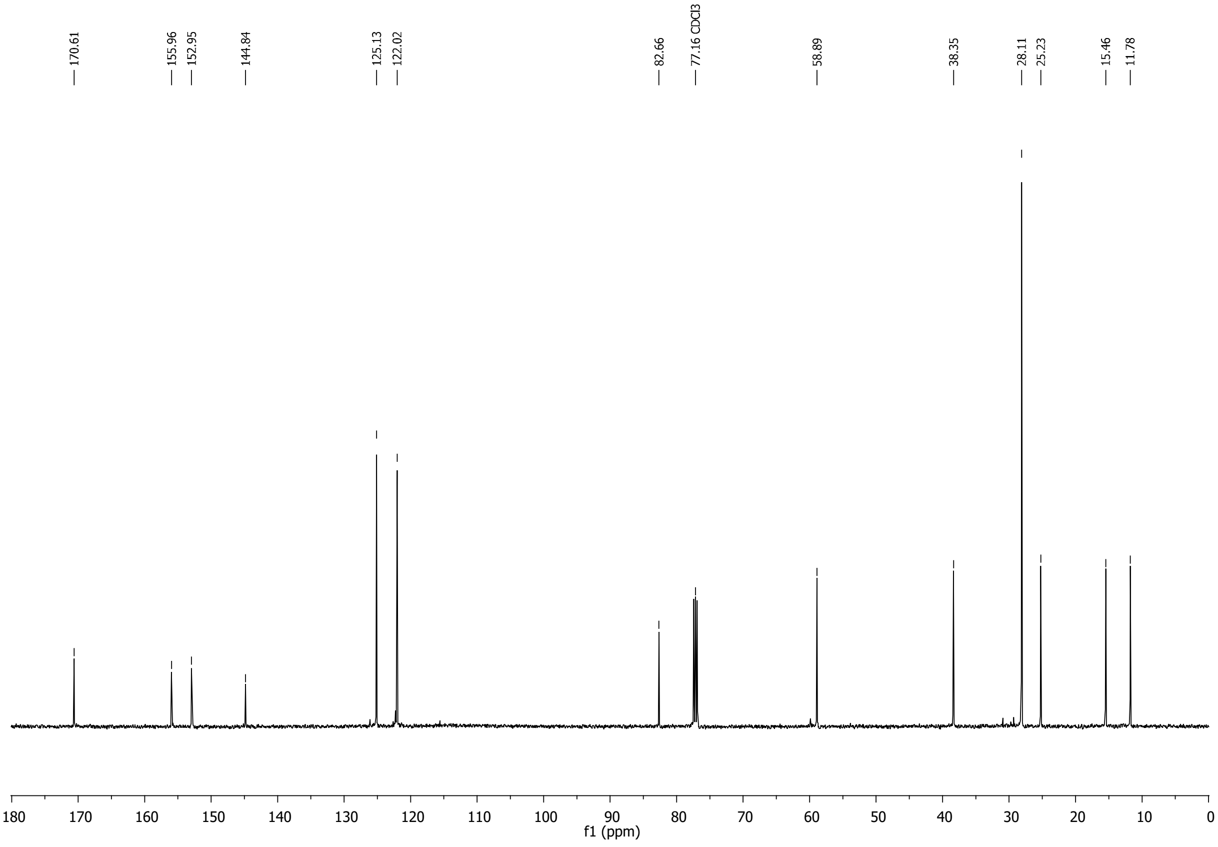

Supplementary Figure S8. *tert-butyl 2-(((4-(3-chloropropyl)phenoxy)carbonyl)amino)-3-methylpentanoate (**3**)*: ^{1}H-NMR (500 MHz, CDCl3) and ^{13}C-NMR (126 MHz, CDCl3)

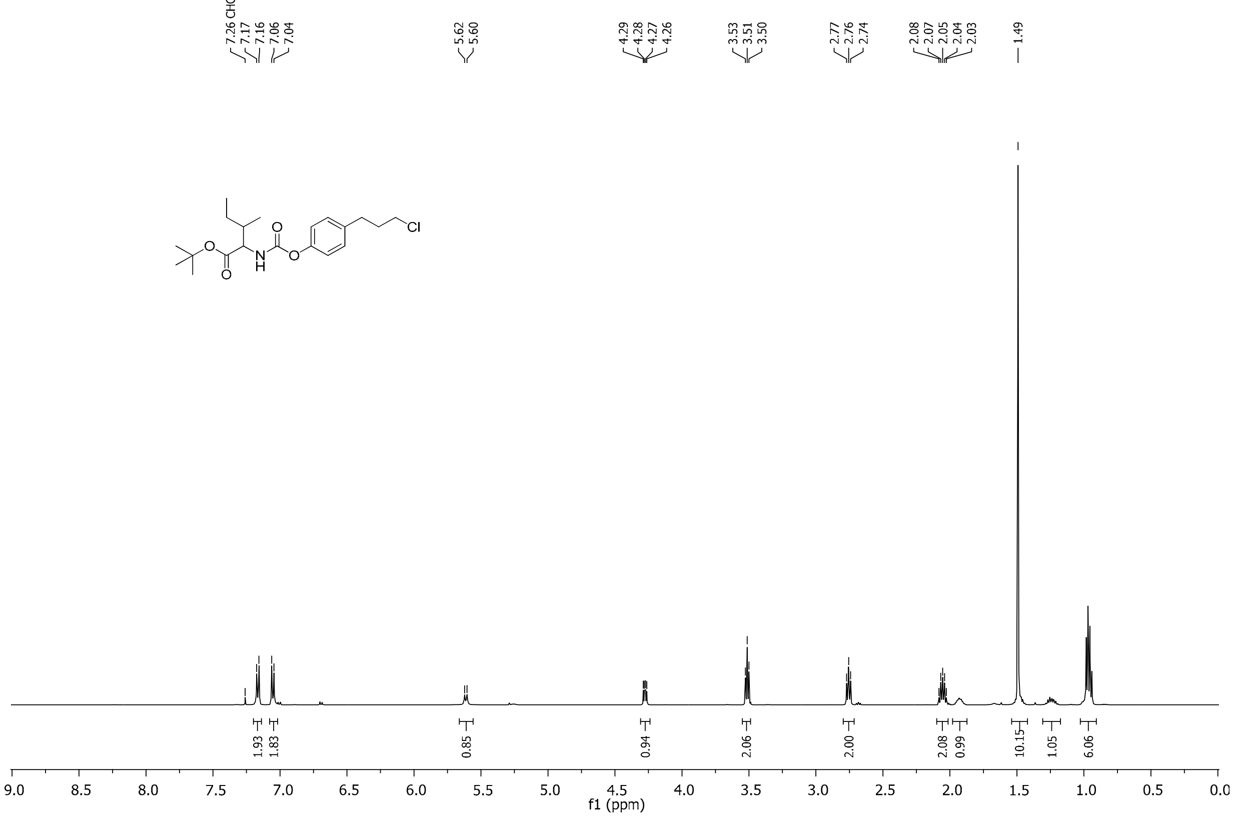

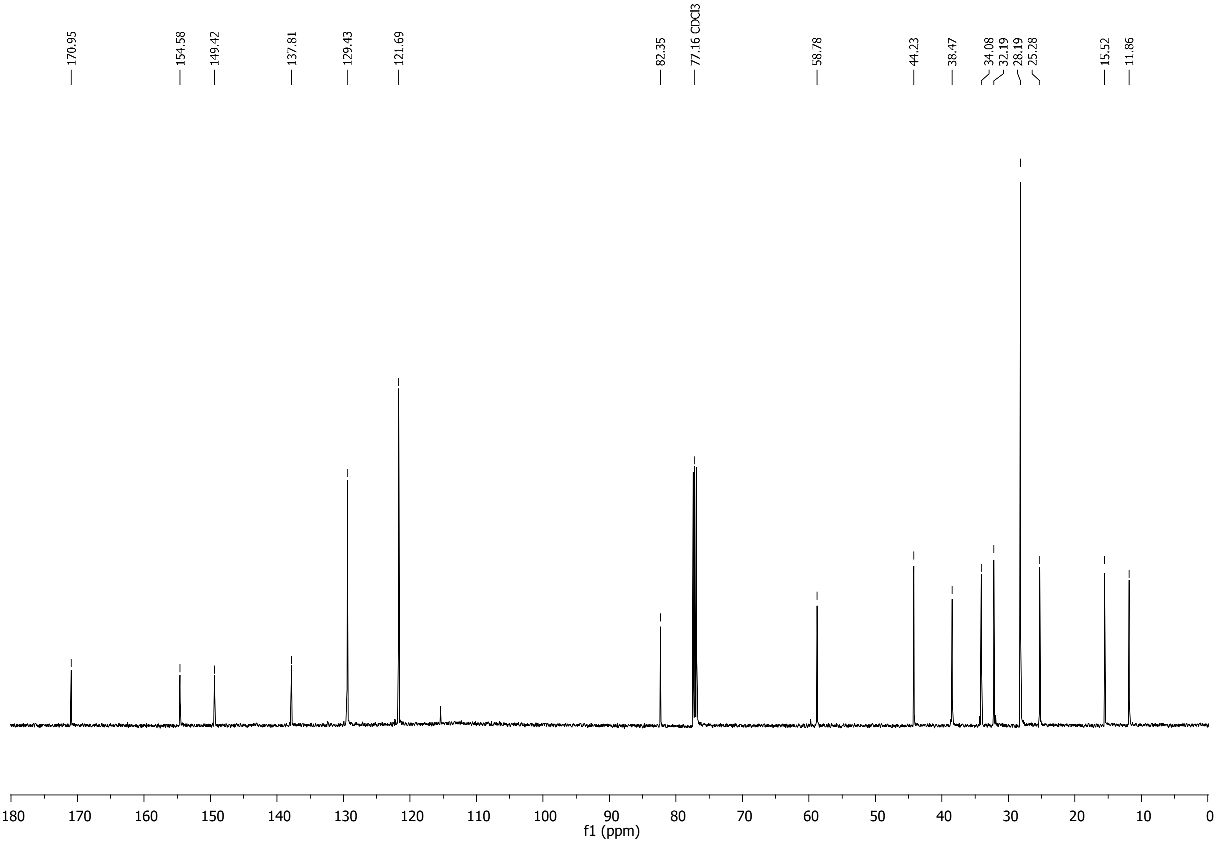

Supplementary Figure S9. *tert-butyl 2-(((4-(3-iodopropyl)phenoxy)carbonyl)amino)-3-methylpentanoate (**4**)*: ^{1}H-NMR (500 MHz, CDCl3) and ^{13}C-NMR (126 MHz, CDCl3)

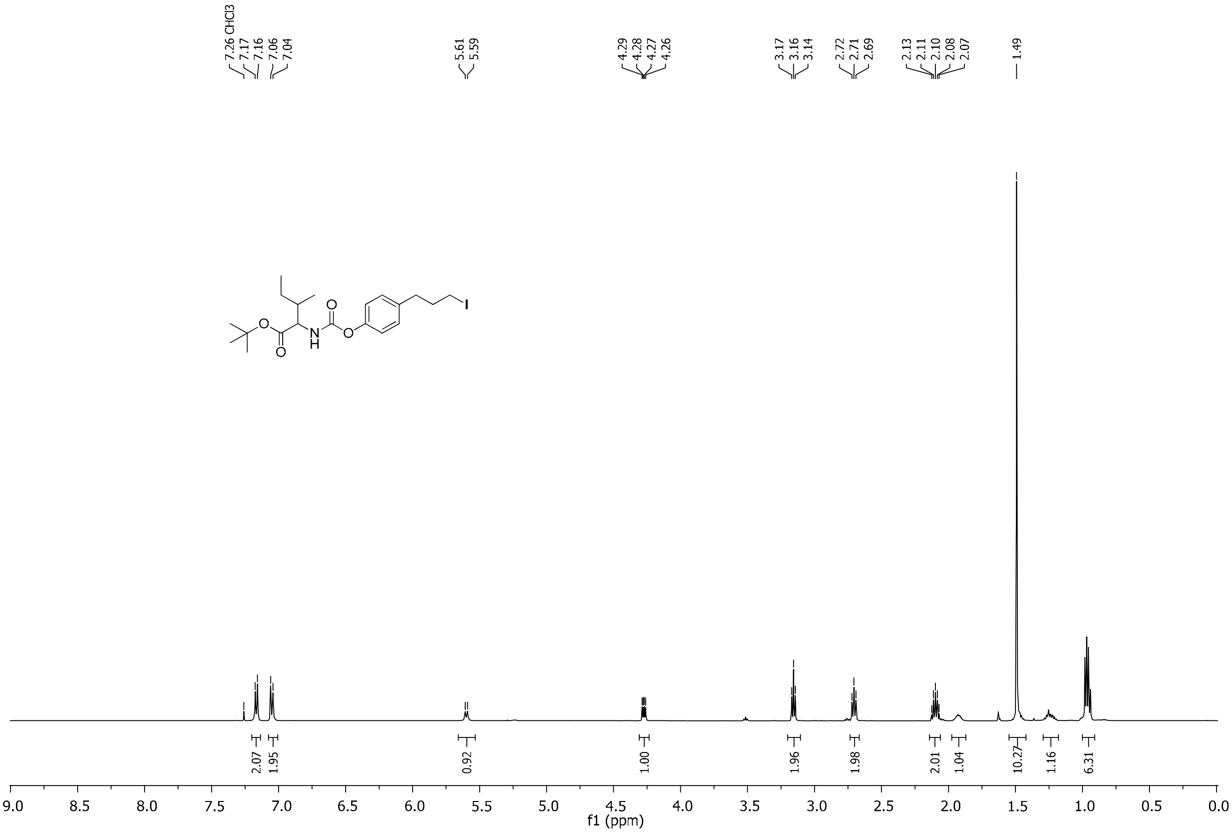

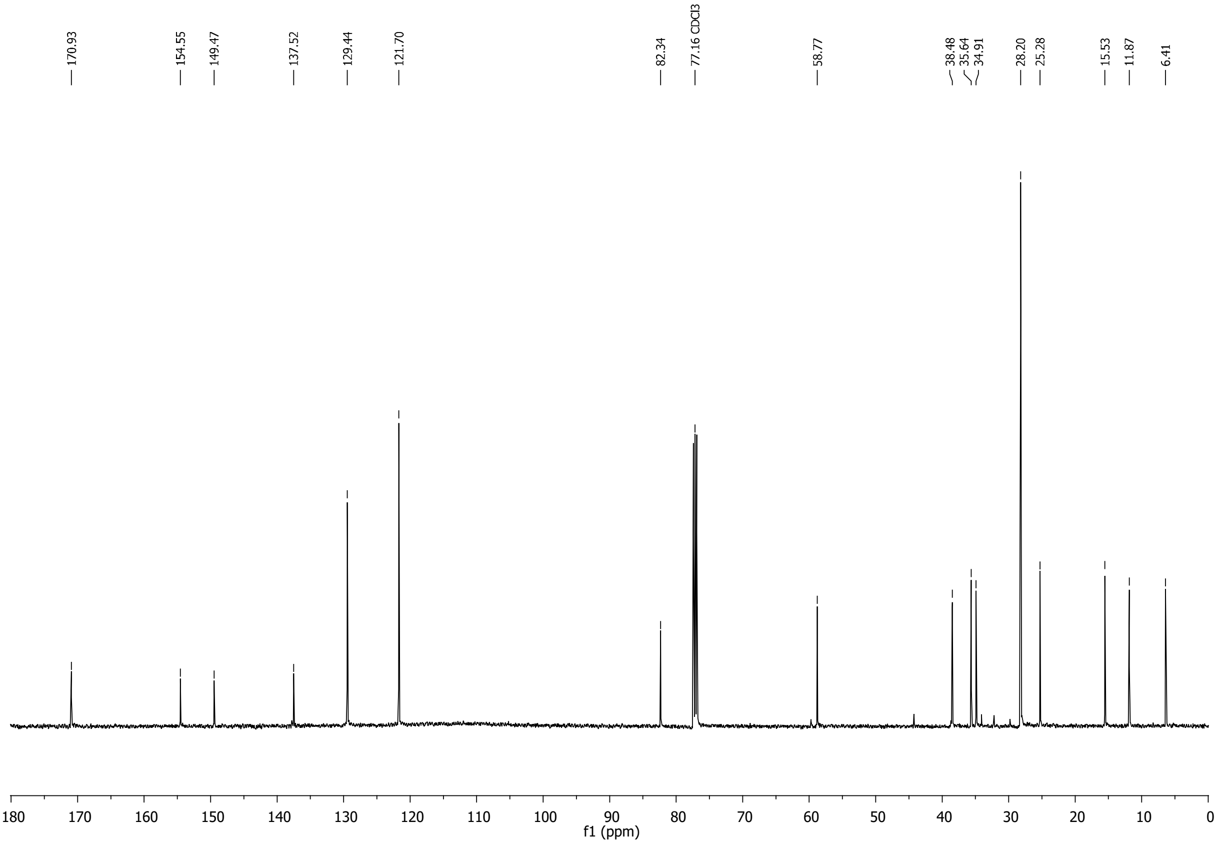

Supplementary Figure S10. *4-hydroxy-7H-furo[3,2-g]chromen-7-one (**5**)*: ^{1}H-NMR (500 MHz, DMSO-*d*$_6$) and ^{13}C-NMR (126 MHz, DMSO-*d*$_6$)

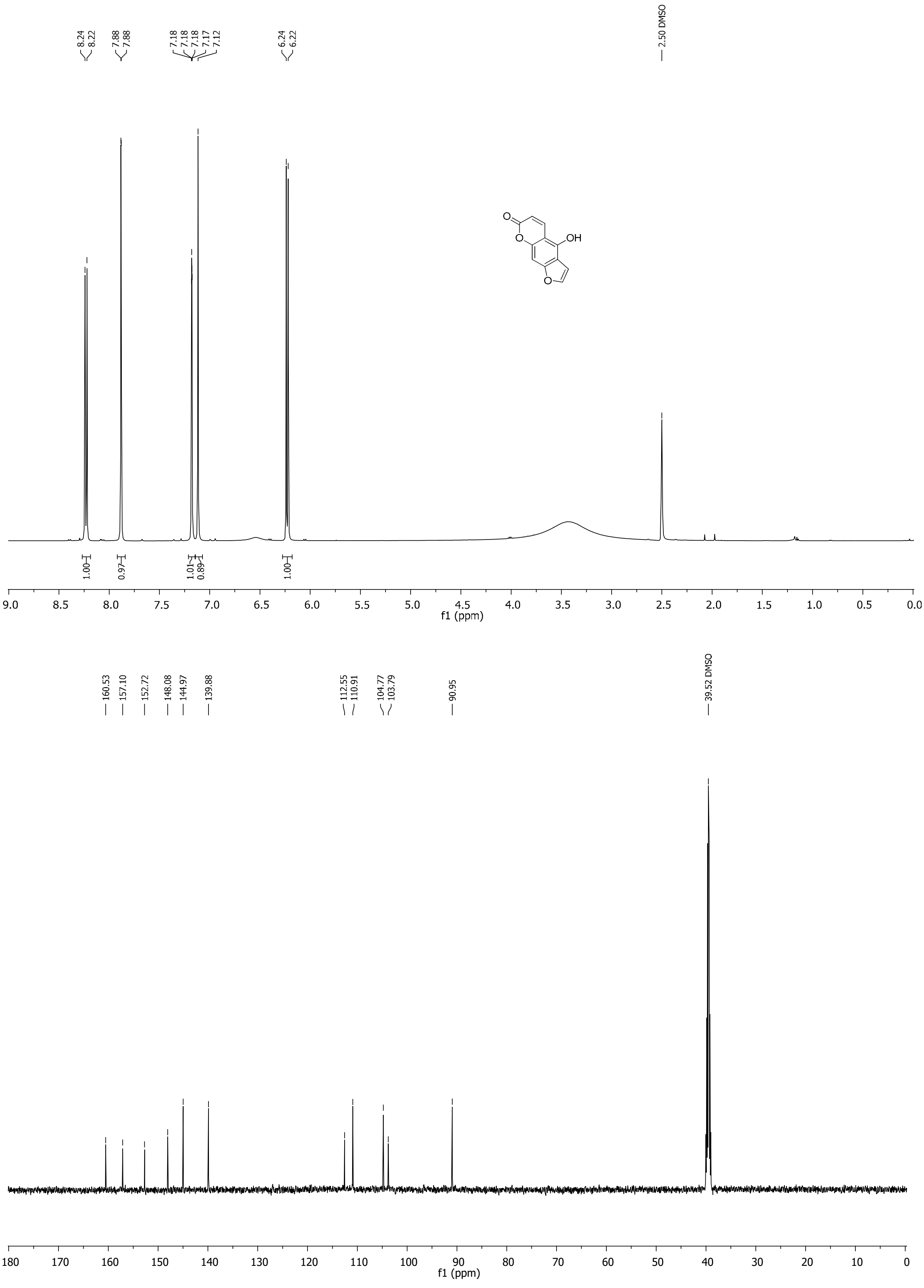

Supplementary Figure S11. *4-(4-chlorobutoxy)-7H-furo[3,2-g]chromen-7-one (**6**)*: ^{1}H-NMR (500 MHz, CDCl3) and ^{13}C-NMR (126 MHz, CDCl3)

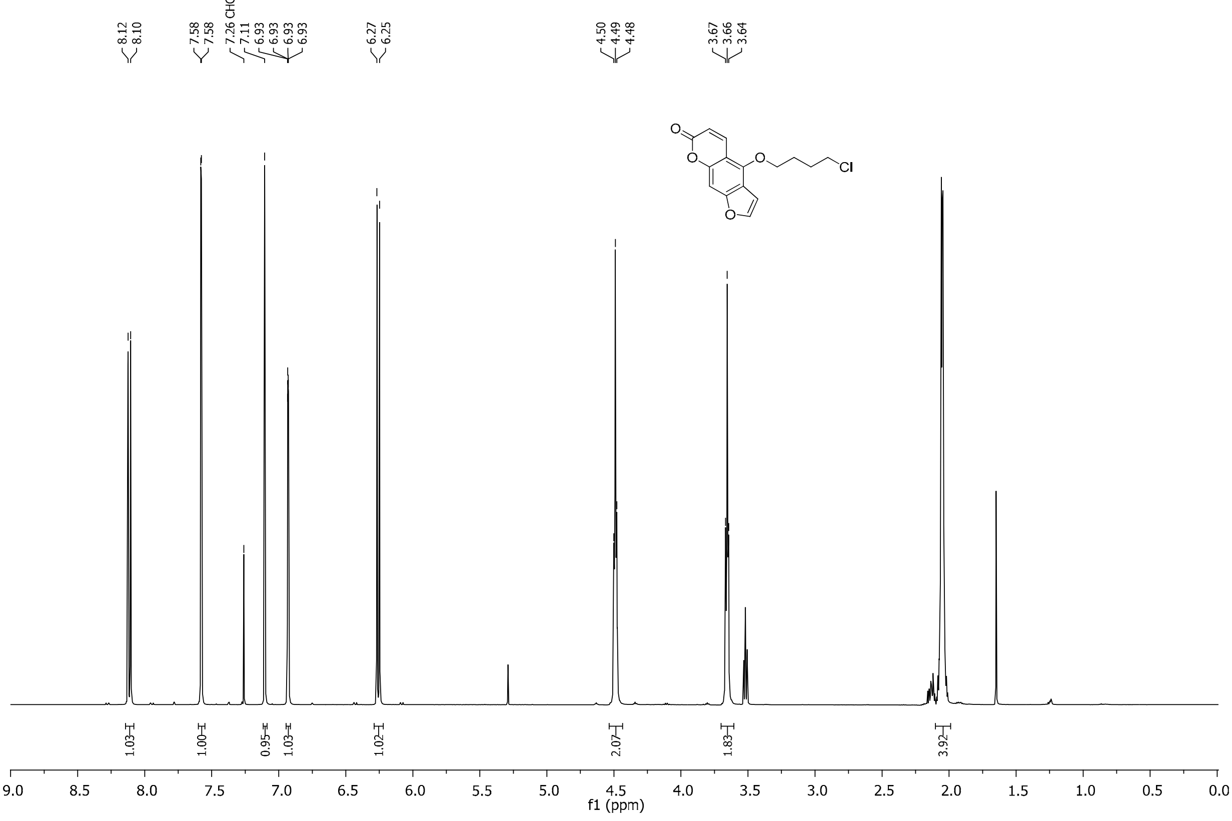

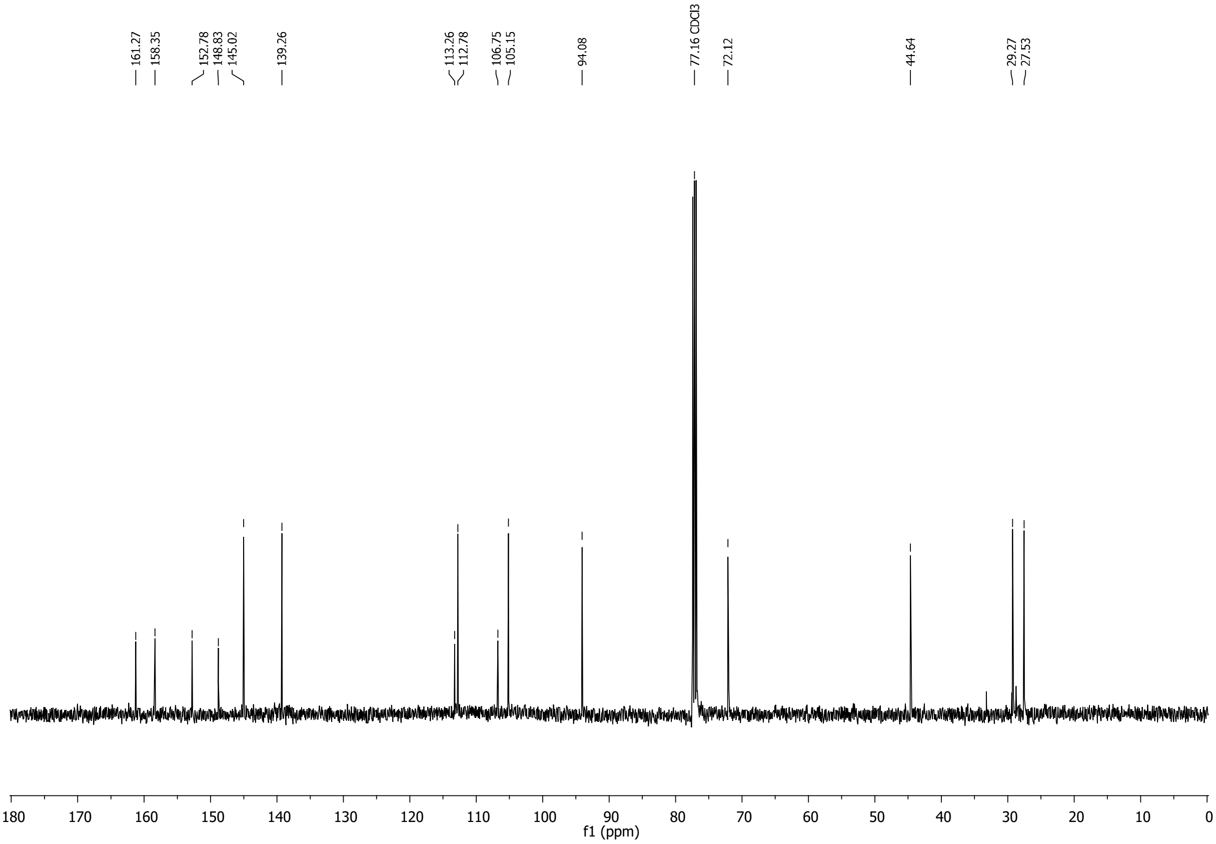

Supplementary Figure S12. *4-(4-iodobutoxy)-7H-furo[3,2-g]chromen-7-one (**7**)*: ^{1}H-NMR (500 MHz, CDCl3) and ^{13}C-NMR (126 MHz, CDCl3)

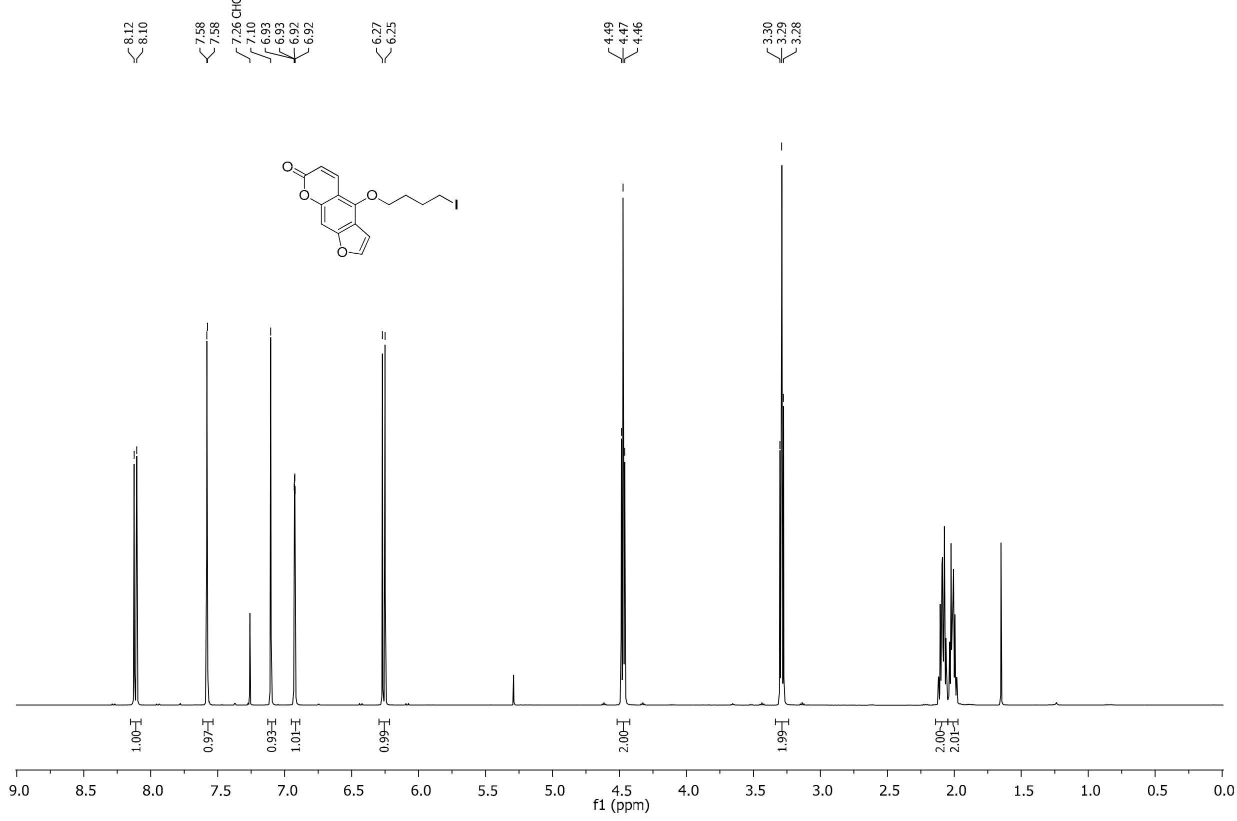

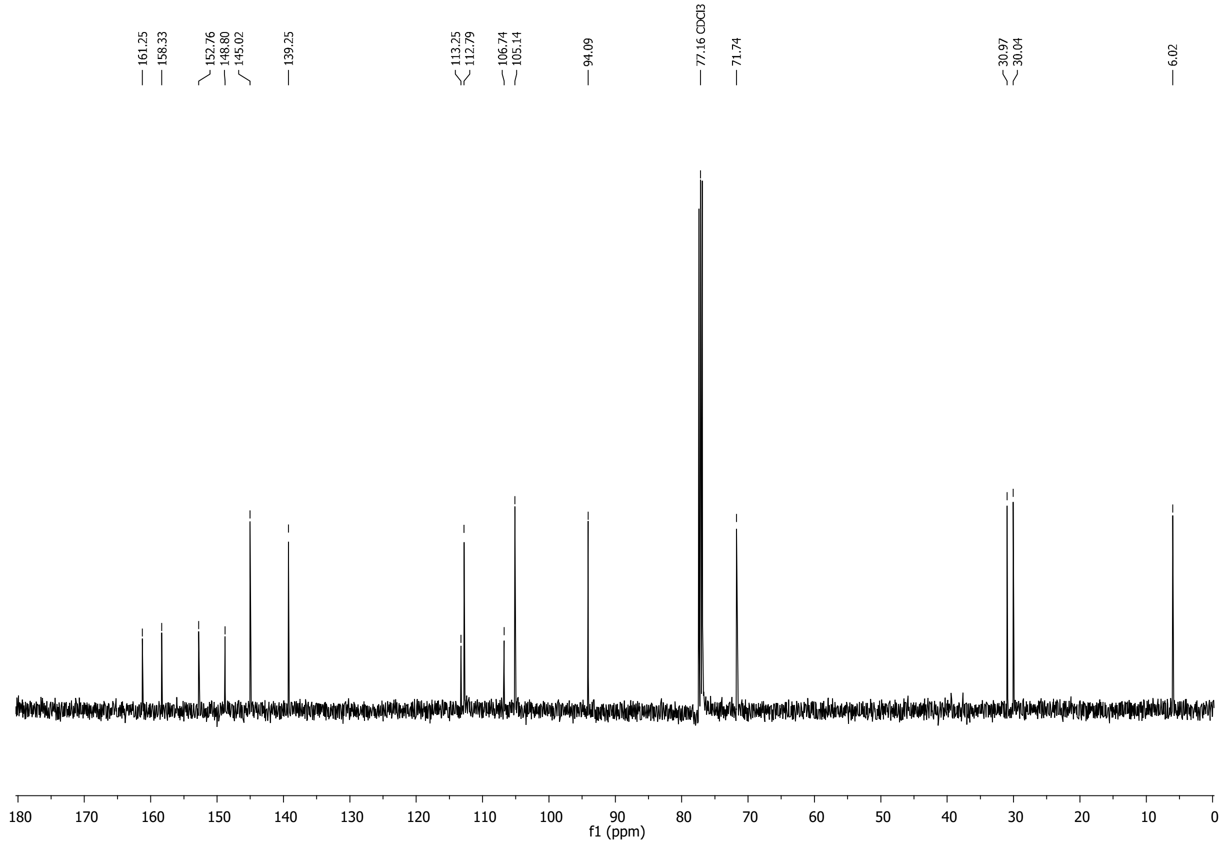

Supplementary Figure S13. *4-(4-(4-(3-hydroxypropyl)phenoxy)butoxy)-7H-furo[3,2-g]chromen-7-one (**8**)*: ^{1}H-NMR (400 MHz, CDCl3) and ^{13}C-NMR (101 MHz, CDCl3)

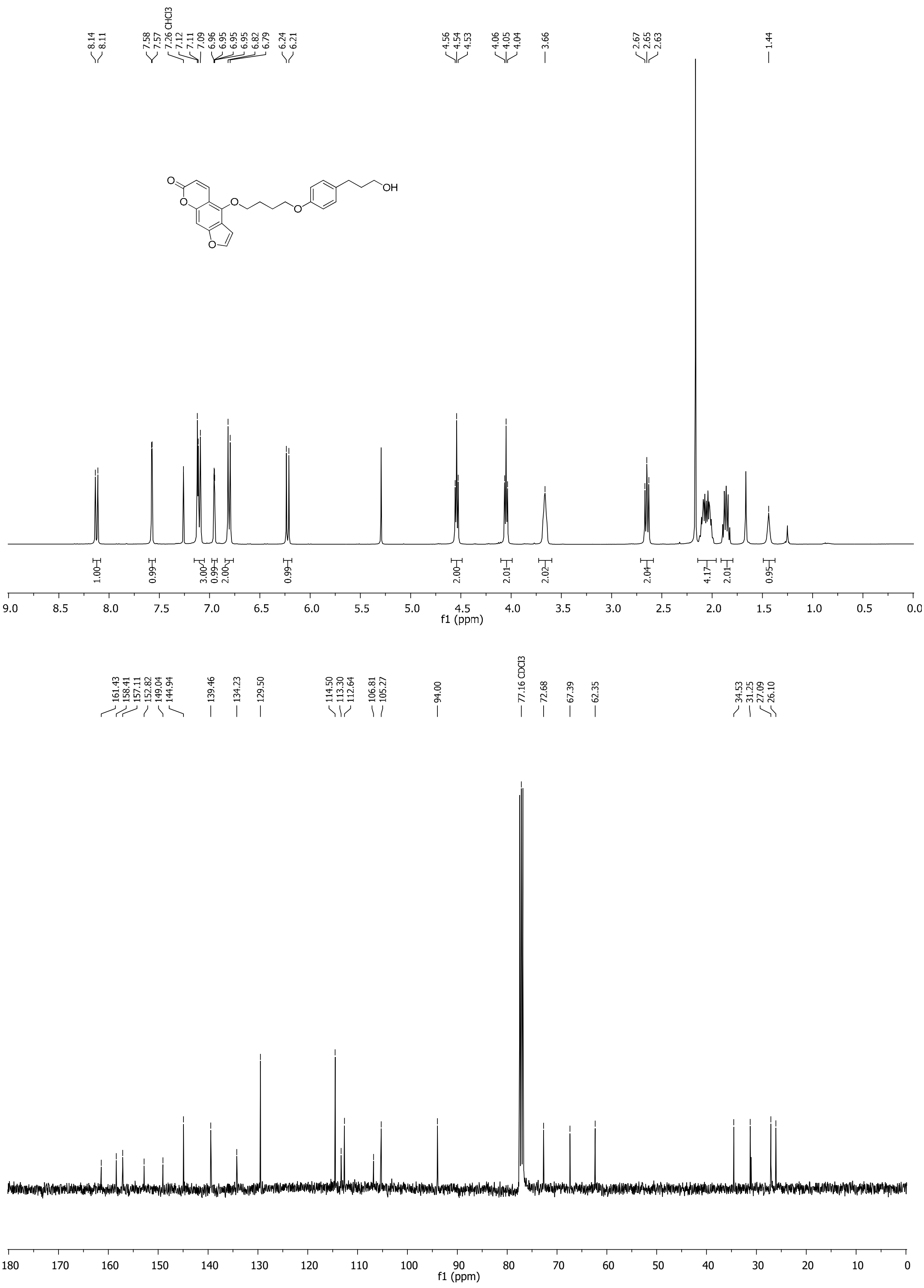

Supplementary Figure S14. *4-(4-(4-(3-chloropropyl)phenoxy)butoxy)-7H-furo[3,2-g]chromen-7-one (**9**)*: ^{1}H-NMR (500 MHz, CDCl3) and ^{13}C-NMR (126 MHz, CDCl3)

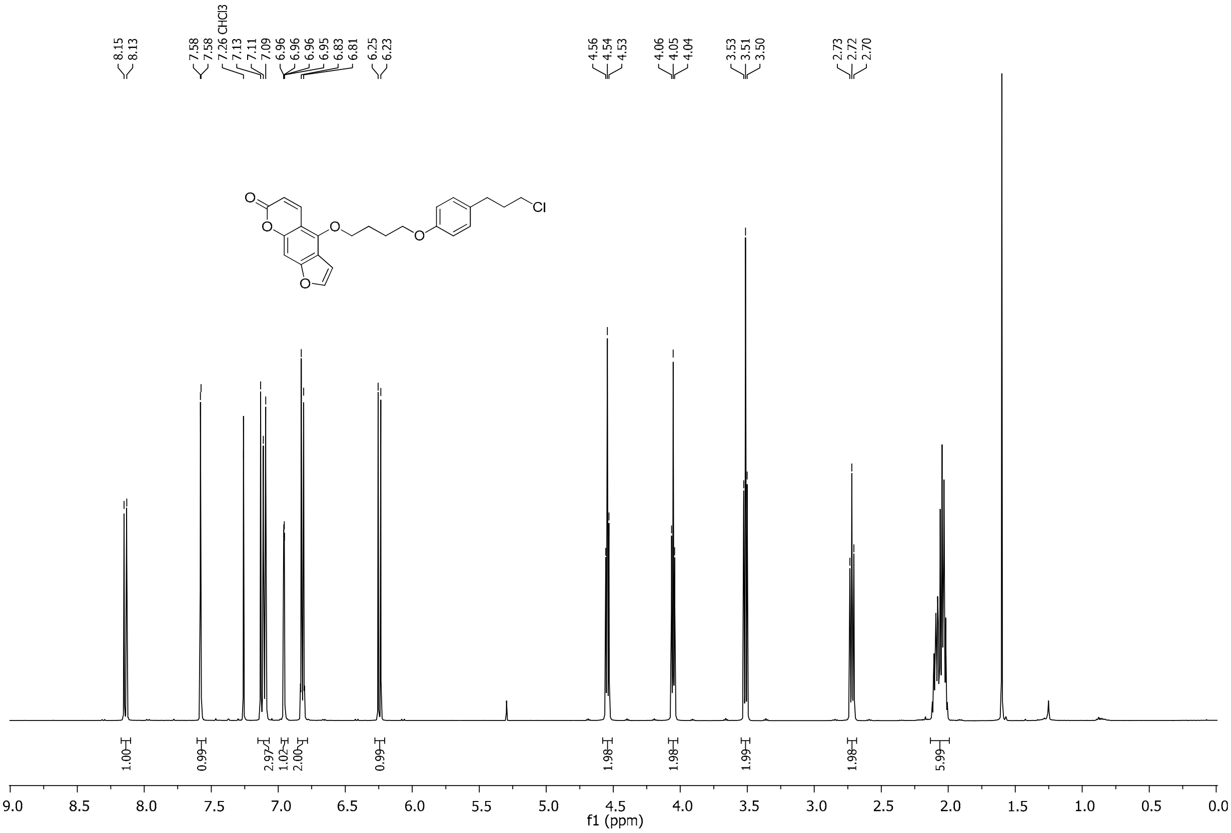

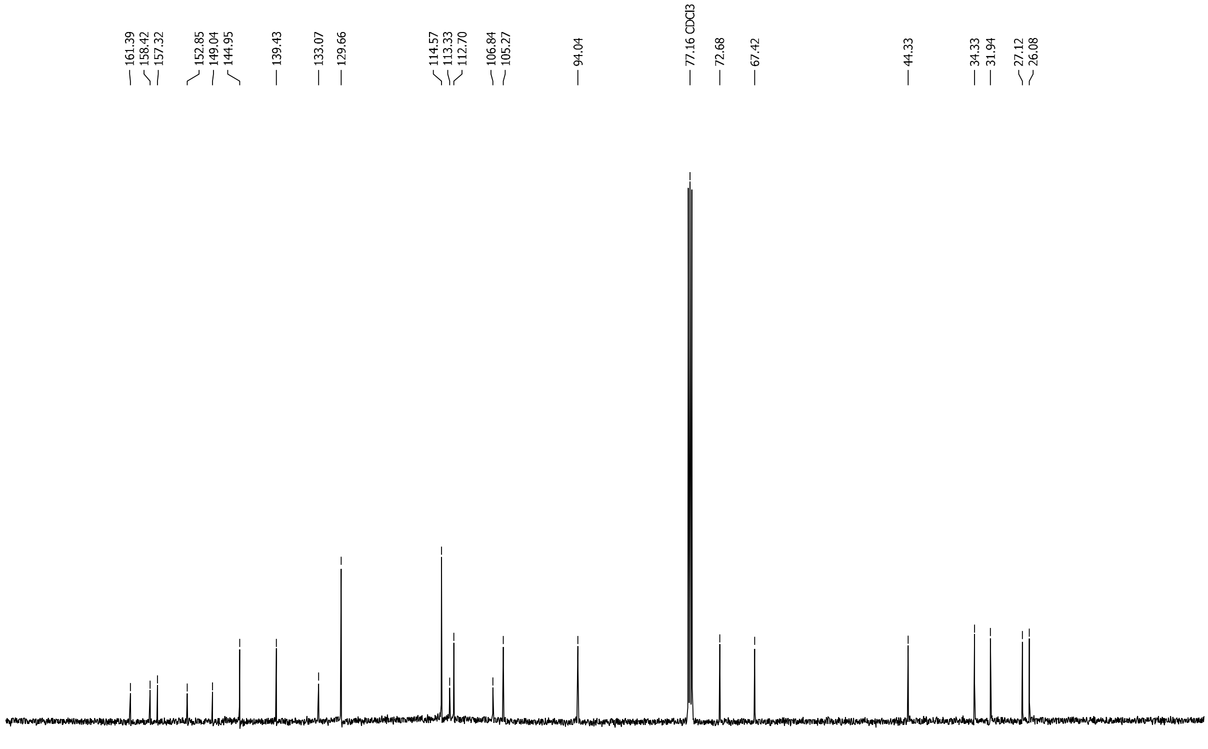

Supplementary Figure S15. *4-(4-(4-(3-iodopropyl)phenoxy)butoxy)-7H-furo[3,2-g]chromen-7-one* (***10***): ^{1}H-NMR (400 MHz, CDCl3) and ^{13}C-NMR (101 MHz, CDCl3)

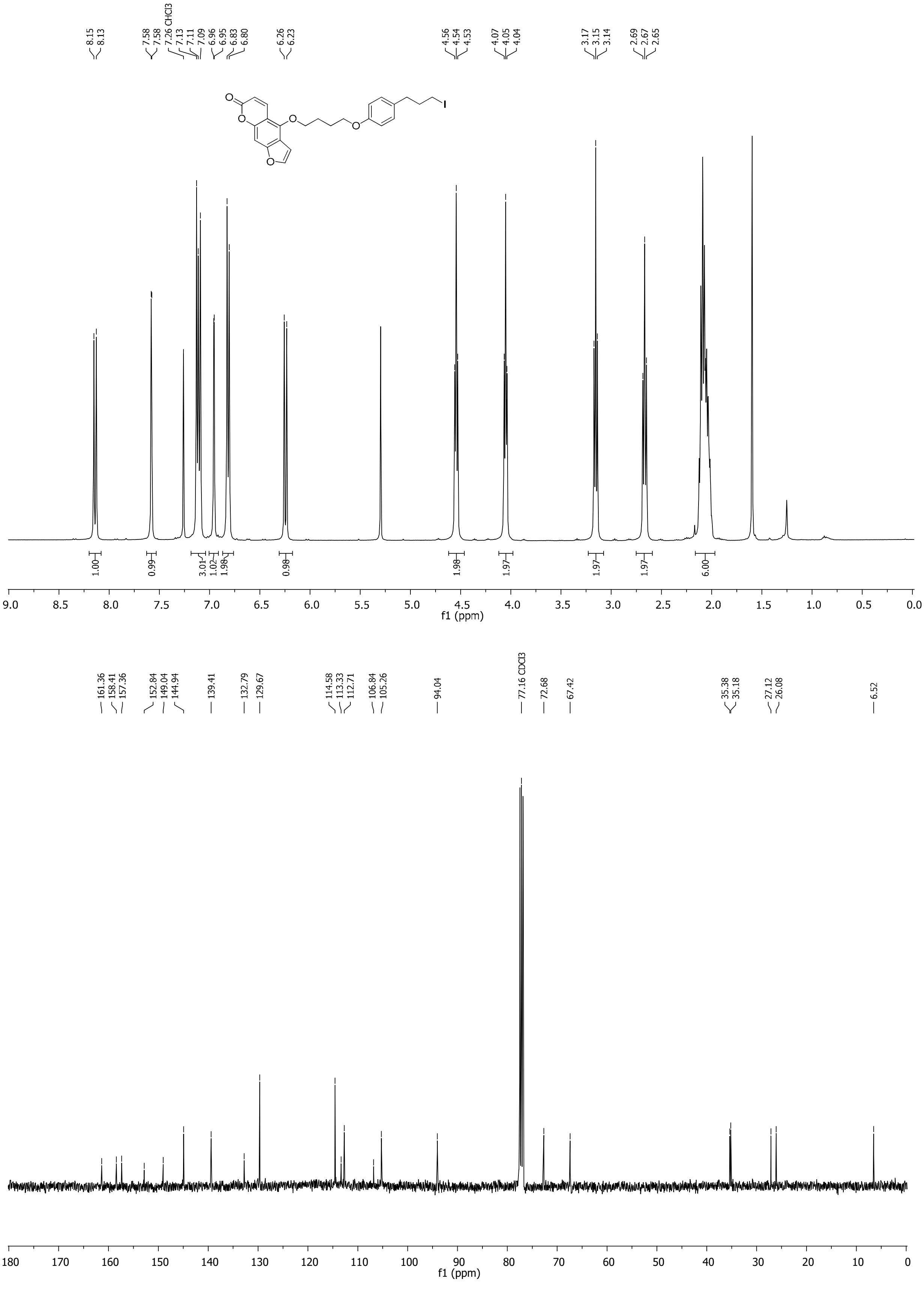

Supplementary Figure S16. *(4-hydroxyphenyl)(3-(4-(4-((7-oxo-7H-furo[3,2-g]chromen-4-yl)oxy)butoxy)phenyl)propyl) diphenylphosphonium iodide (**11**)*: ^{1}H-NMR (400 MHz, CDCl3) and ^{13}C-NMR (101 MHz, CDCl3)

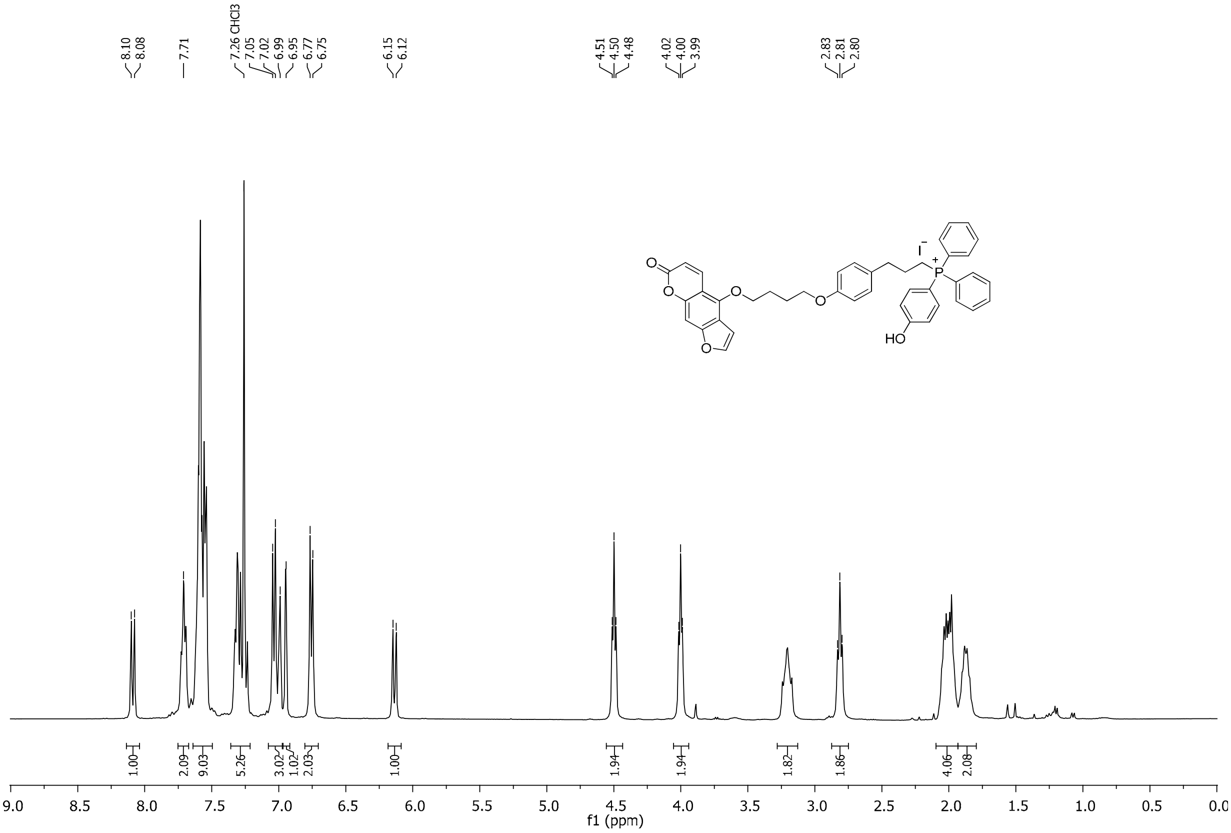

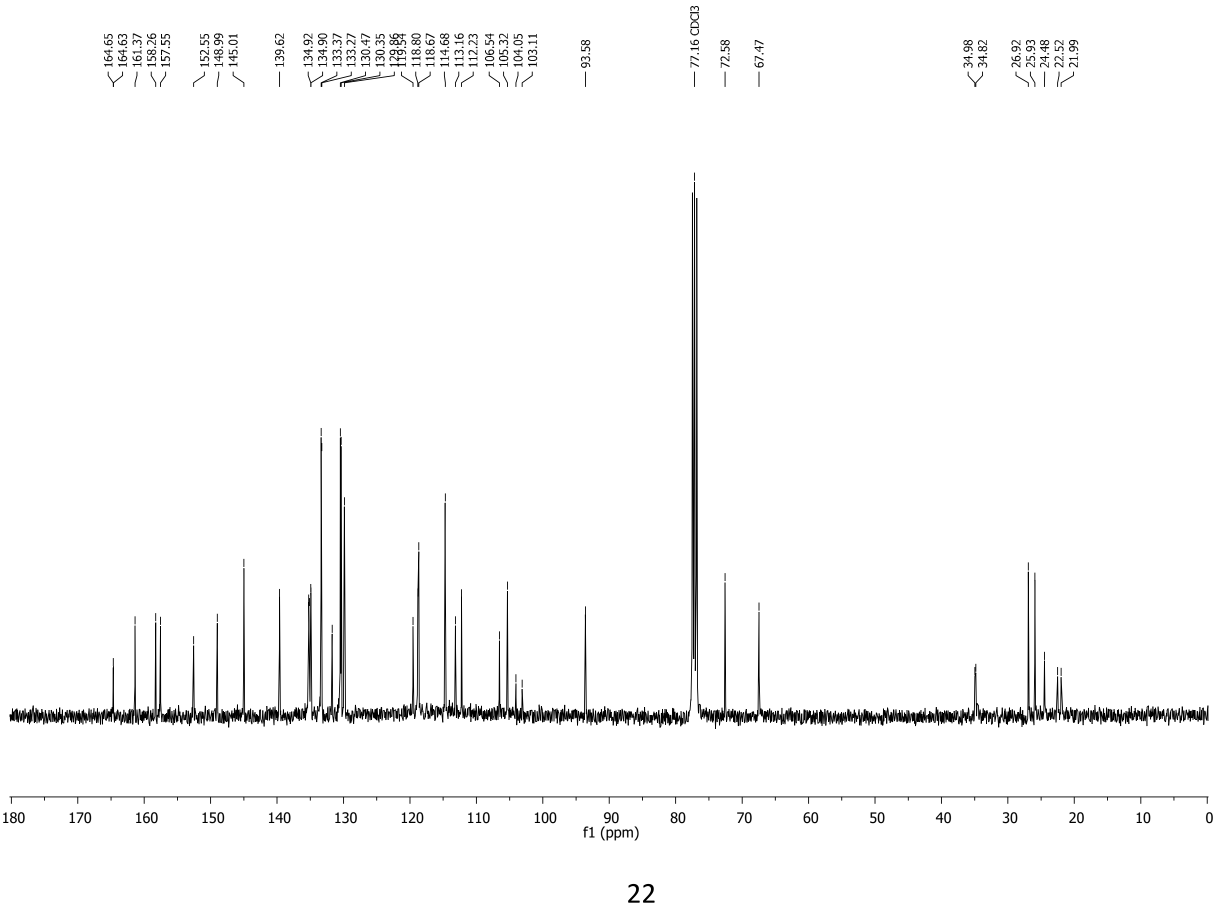

Supplementary Figure S17. *(4-(3-(4-(((1-(tert-butoxy)-3-methyl-1-oxopentan-2-yl)carbamoyl)oxy)phenyl)propoxy)phenyl)(3-(4-(4-((7-oxo-7H-furo[3,2-g]chromen-4-yl)oxy)butoxy)phenyl)propyl)diphenylphosphonium 2,2,2-trifluoroacetate (**12**)*: ^{1}H-NMR (500 MHz, DMSO-*d*$_6$) and ^{13}C-NMR (126 MHz, DMSO-*d*$_6$)

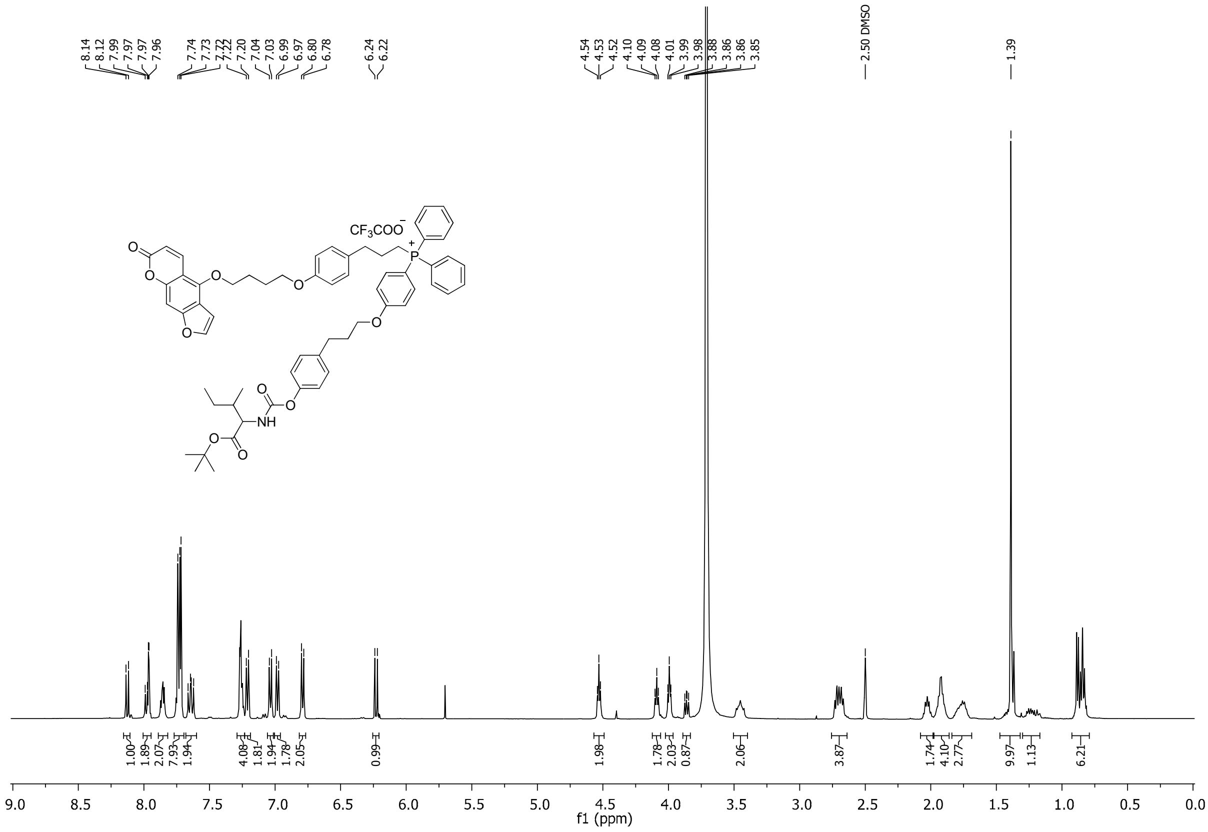

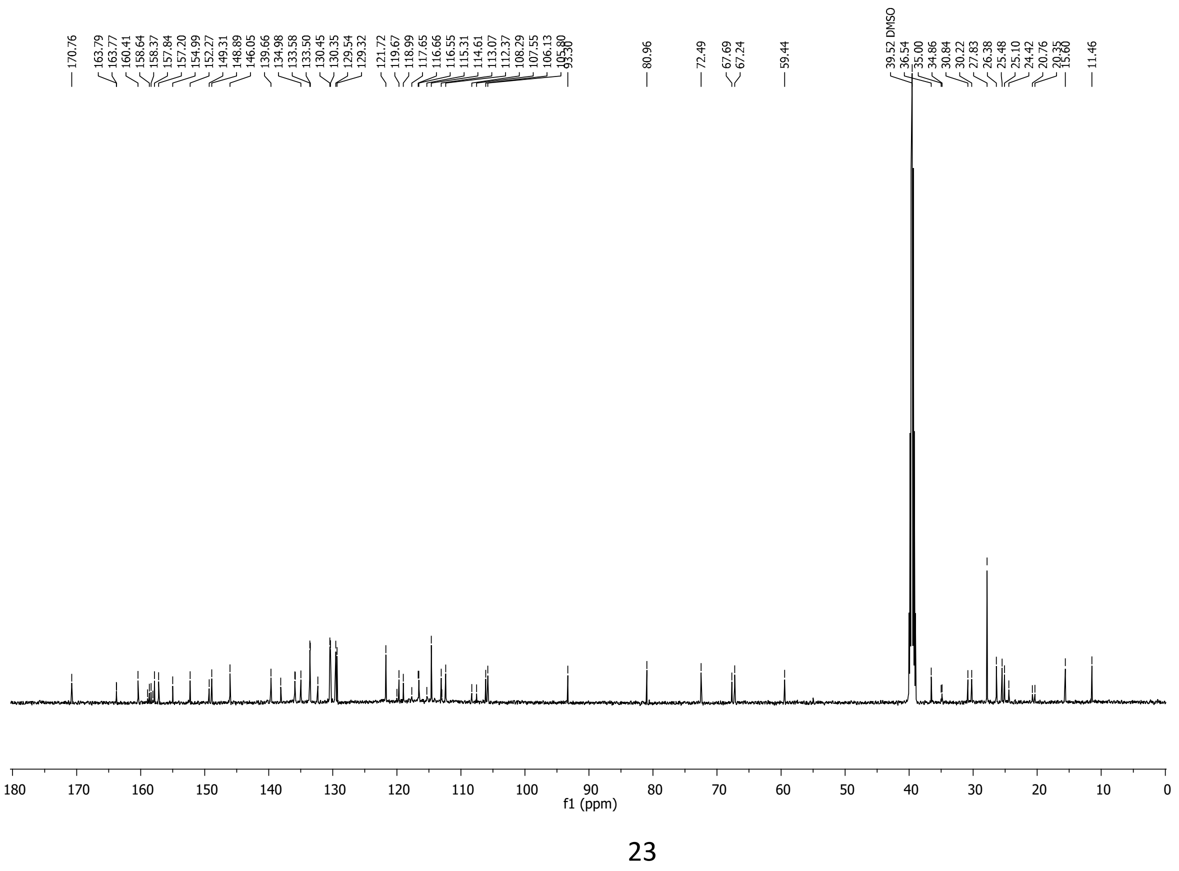

Supplementary Figure S18. *(4-(3-(4-(((1-carboxy-2-methylbutyl)carbamoyl)oxy)phenyl)propoxy)phenyl)(3-(4-(4-((7-oxo-7H-furo[3,2-g]chromen-4-yl)oxy)butoxy)phenyl)propyl)diphenylphosphonium 2,2,2-trifluoroacetate (**PAPTPL-I**)*: ^{1}H-NMR (500 MHz, CD_2Cl_2) and ^{13}C-NMR (101 MHz, CD_2Cl_2)

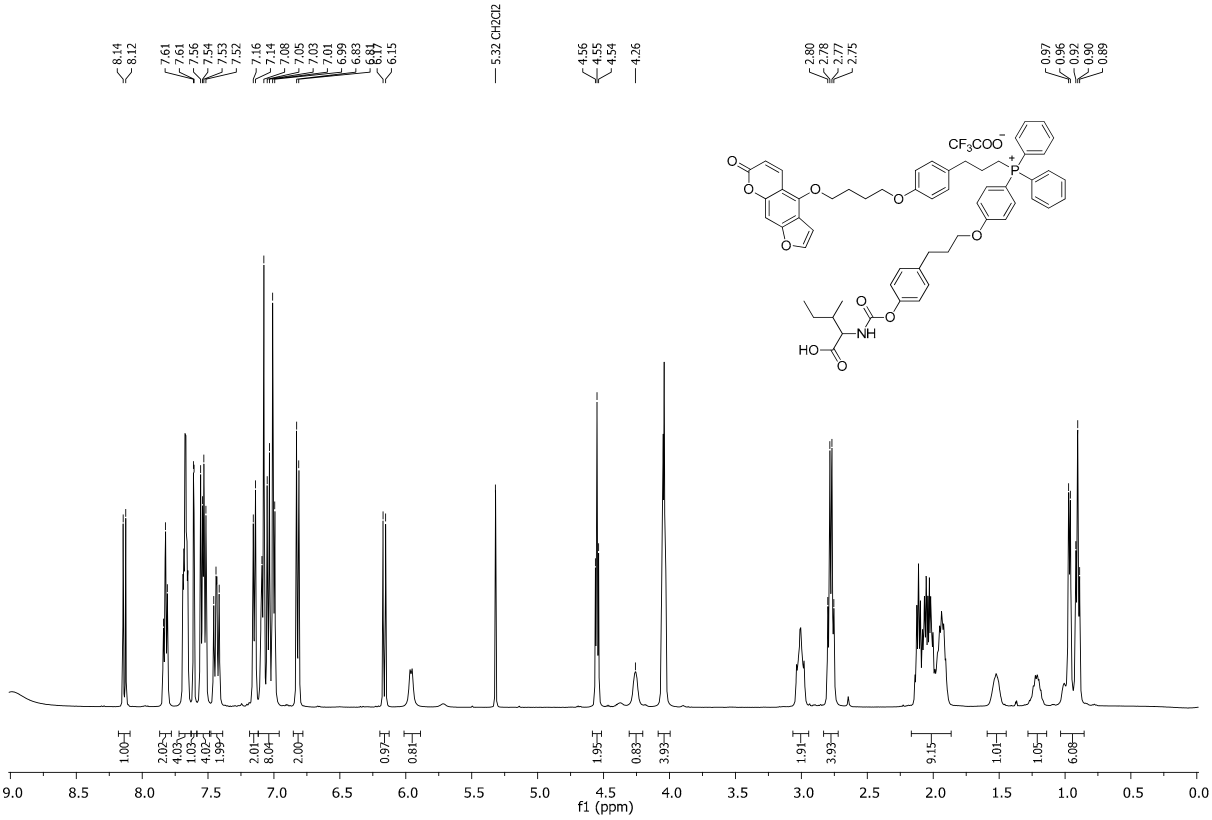

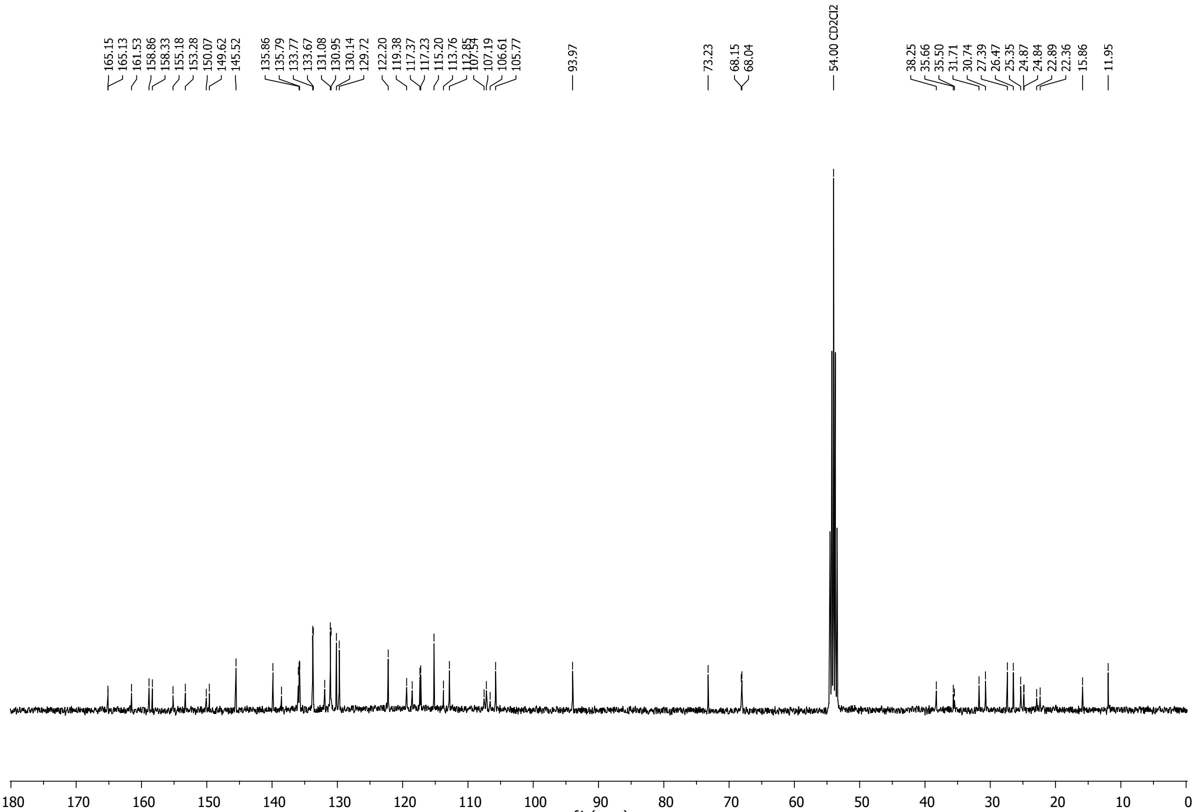

Supplementary Figure S19. *(4-(3-(4-hydroxyphenyl)propoxy)phenyl)(3-(4-(4-((7-oxo-7H-furo[3,2-g]chromen-4-yl)oxy)butoxy)phenyl) propyl)diphenylphosphonium 2,2,2-trifluoroacetate (**PAPTPL**)*: ^{1}H-NMR (500 MHz, DMSO-d_6) and ^{13}C-NMR (126 MHz, DMSO-d_6)

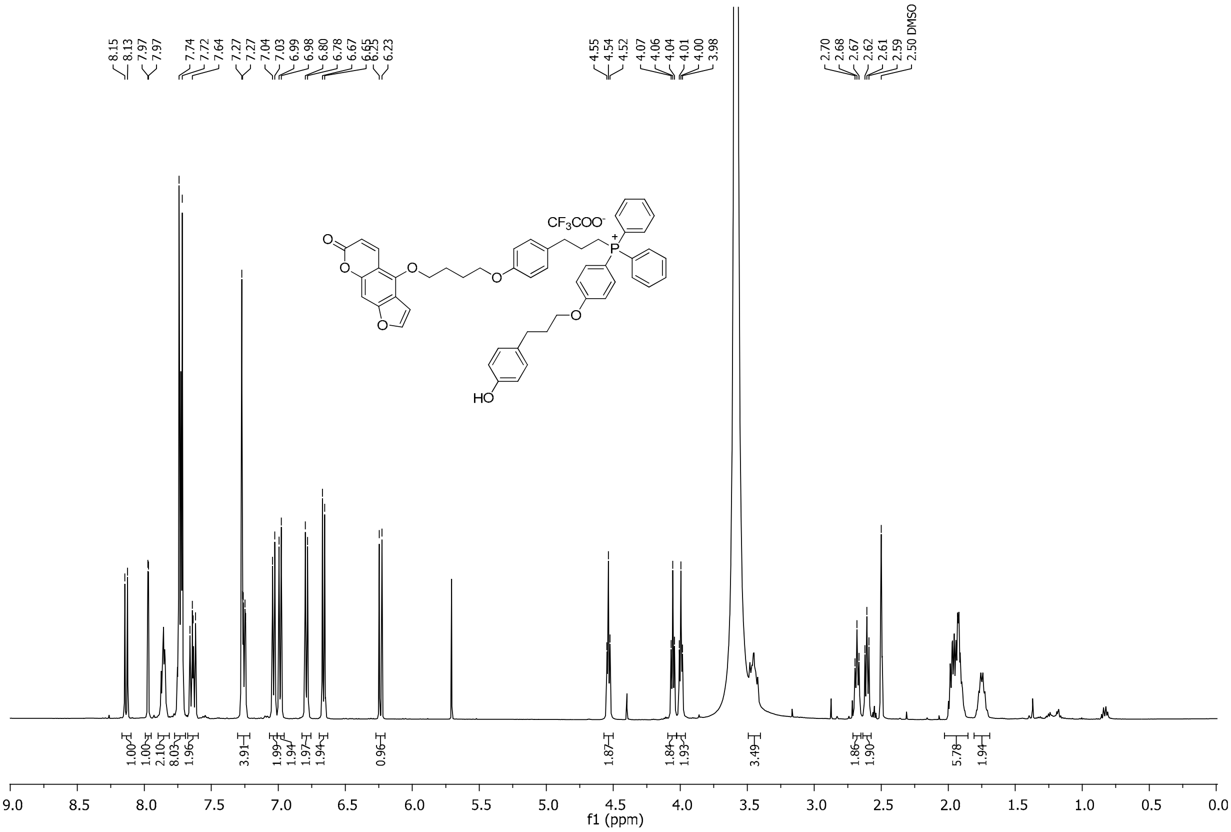

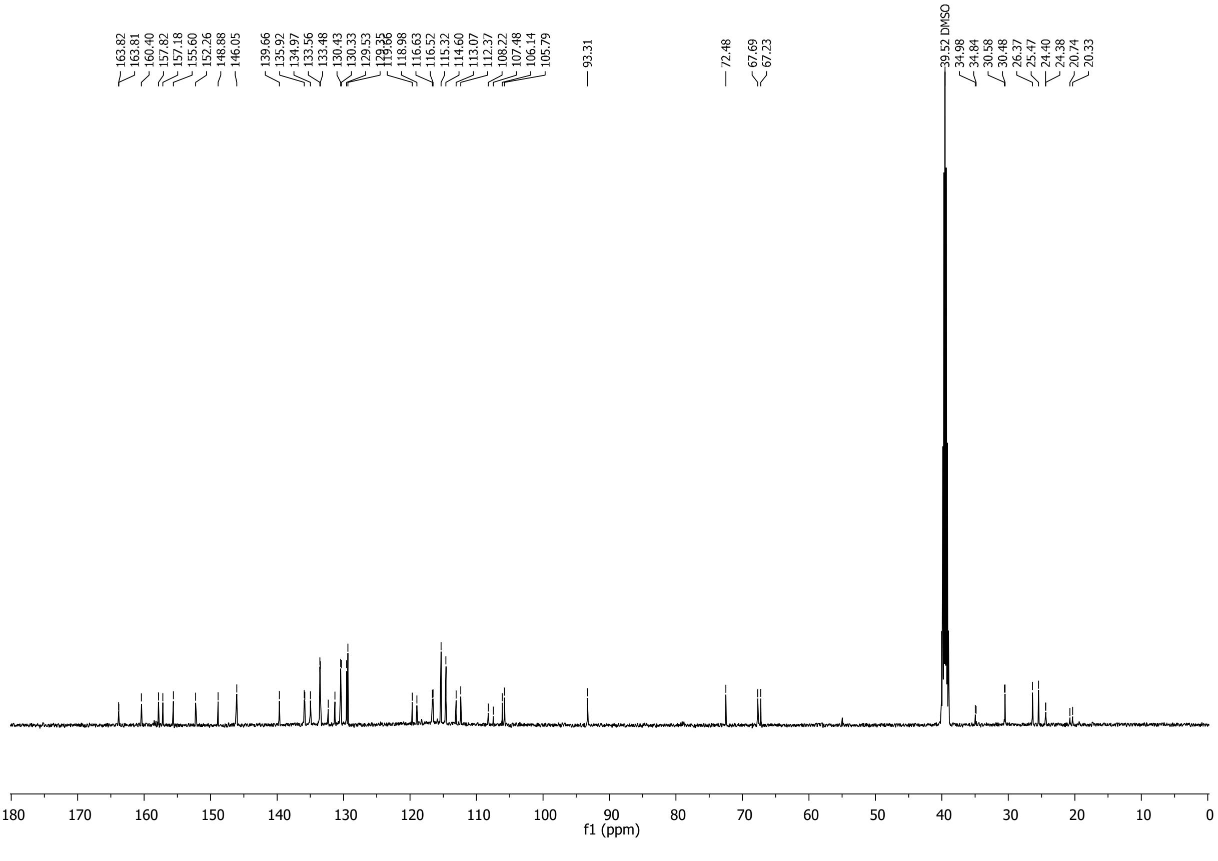

Supplementary Figure S20. *HPLC-UV chromatogram (recorded at 312 nm) of a 25 µM solution of TAT-PAPTP in ACN + 0.1% TFA. Insert: UV-absorption spectrum recorded at the retention time of TAT-PAPTP.*

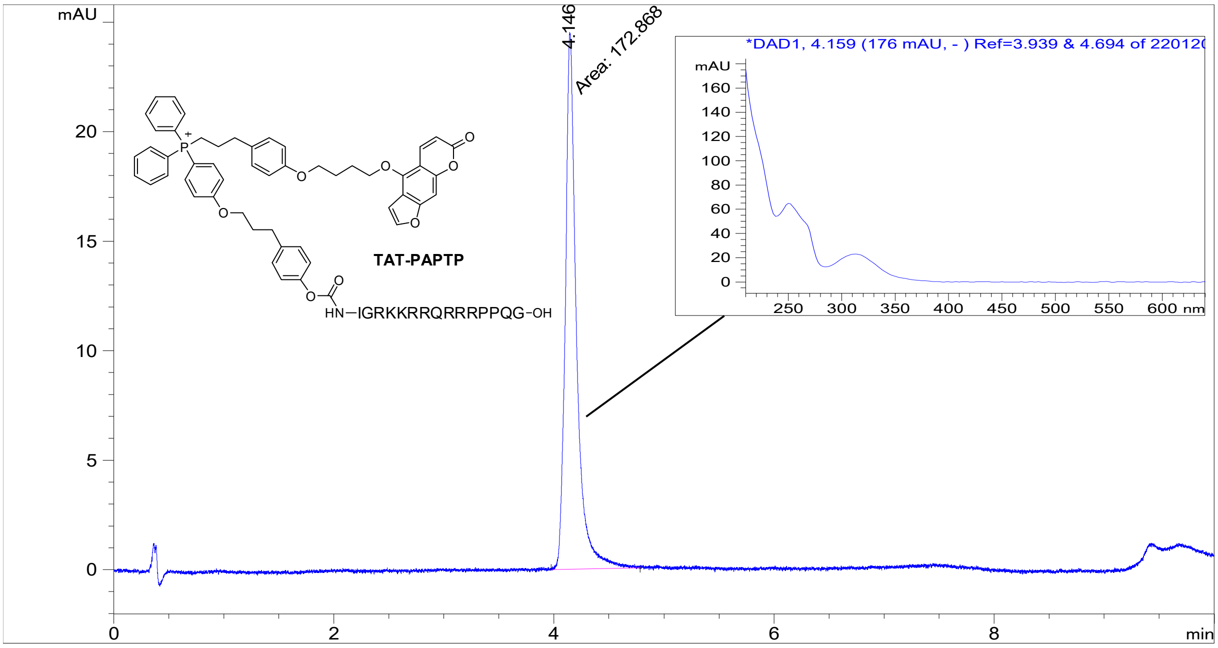

Supplementary Figure S21. *HPLC-UV chromatogram (recorded at 312 nm) of a 25 µM solution of An2-PAPTP in ACN + 0.1% TFA. Insert: UV-absorption spectrum recorded at the retention time of An2-PAPTP.*

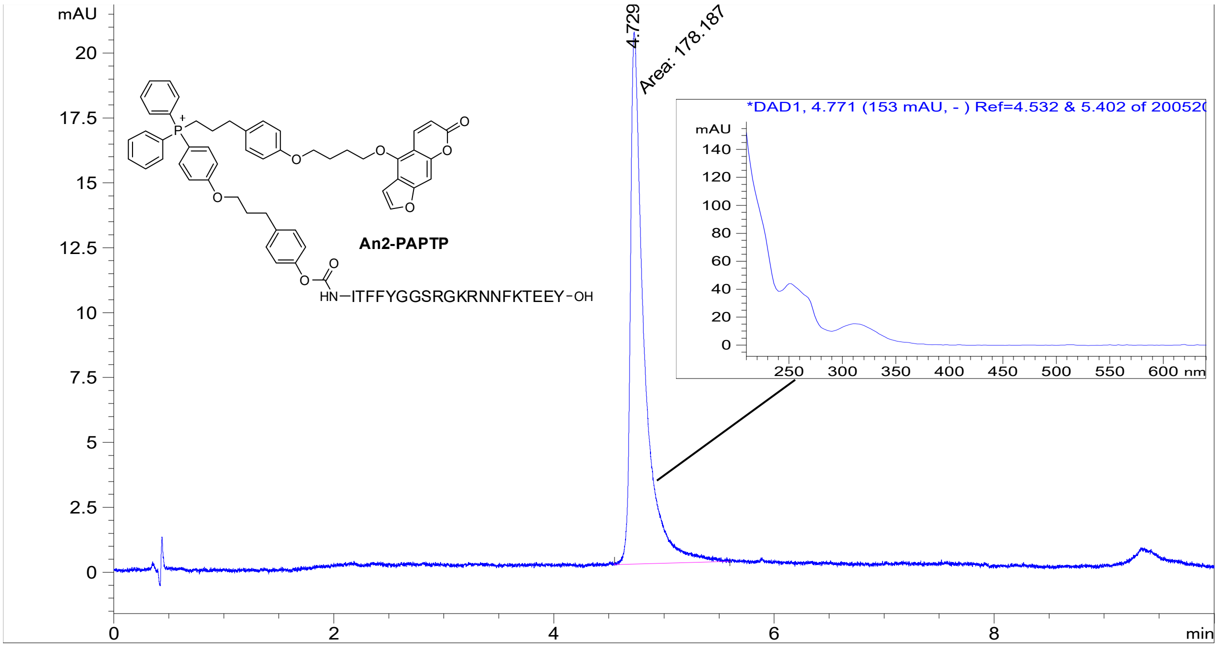

Supplementary Figure S22. *HPLC-UV chromatogram (recorded at 312 nm) of a 25 µM solution of PAPTPL in ACN + 0.1% TFA. Insert: UV-absorption spectrum recorded at the retention time of PAPTPL.*

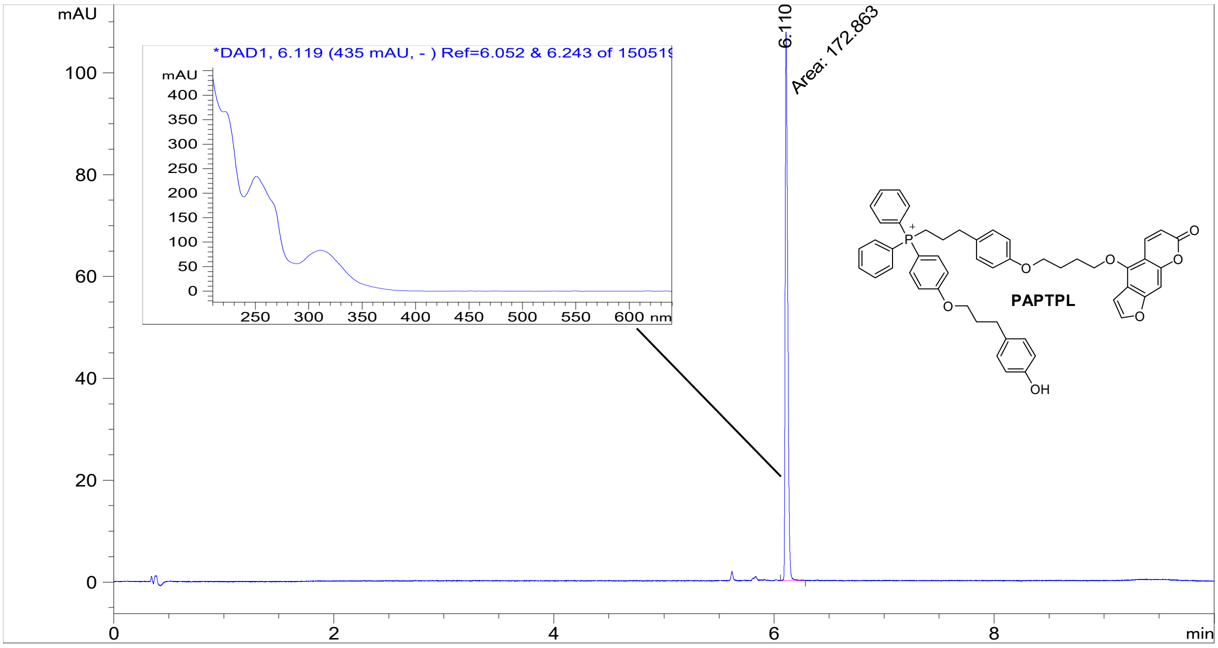

Supplement: Supplementary file 1 [file pharmaceuticals-14-00129-s001.pdf]
